# Dielectric Barrier Plasma Discharge Exsolution of Nanoparticles at Room Temperature and Atmospheric Pressure

**DOI:** 10.1002/advs.202402235

**Published:** 2024-07-04

**Authors:** Atta ul Haq, Fiorenza Fanelli, Leonidas Bekris, Alex Martinez Martin, Steve Lee, Hessan Khalid, Cristian D. Savaniu, Kalliopi Kousi, Ian S. Metcalfe, John T. S. Irvine, Paul Maguire, Evangelos I. Papaioannou, Davide Mariotti

**Affiliations:** ^1^ School of Engineering Ulster University Belfast BT37 0QB UK; ^2^ Institute of Nanotechnology (NANOTEC) National Research Council (CNR) via Orabona 4 Bari 70125 Italy; ^3^ Institute of Chemistry of Organometallic Compounds (ICCOM) National Research Council (CNR) via Orabona 4 Bari 70125 Italy; ^4^ School of Engineering Newcastle University Newcastle upon Tyne NE1 7RU UK; ^5^ School of Physics and Astronomy University of St. Andrews Scotland Fife St. Andrews KY16 9SS UK; ^6^ School of Chemistry University of St. Andrews Scotland Fife St. Andrews KY16 9ST UK; ^7^ School of Chemistry & Chemical Engineering University of Surrey Guildford Surrey GU2 7XH UK; ^8^ Department of Design Manufacturing & Engineering Management University of Strathclyde Glasgow G1 1XJ UK

**Keywords:** catalysis, exsolution, Ni nanoparticles, perovskites, plasma

## Abstract

Exsolution of metal nanoparticles (NPs) on perovskite oxides has been demonstrated as a reliable strategy for producing catalyst‐support systems. Conventional exsolution requires high temperatures for long periods of time, limiting the selection of support materials. Plasma direct exsolution is reported at room temperature and atmospheric pressure of Ni NPs from a model A‐site deficient perovskite oxide (La_0.43_Ca_0.37_Ni_0.06_Ti_0.94_O_2.955_). Plasma exsolution is carried out within minutes (up to 15 min) using a dielectric barrier discharge configuration both with He‐only gas as well as with He/H_2_ gas mixtures, yielding small NPs (<30 nm diameter). To prove the practical utility of exsolved NPs, various experiments aimed at assessing their catalytic performance for methanation from synthesis gas, CO, and CH_4_ oxidation are carried out. Low‐temperature and atmospheric pressure plasma exsolution are successfully demonstrated and suggest that this approach could contribute to the practical deployment of exsolution‐based stable catalyst systems.

## Graphical abstract

Plasma direct exsolution at atmospheric pressures is presented. This groundbreaking method operating at room temperatures swiftly produces nickel nanoparticles on the perovskite oxides surface. These exsolved nanocatalysts with diameters less than 30 nm result in enhanced catalytic reactions in methanation and CO oxidations. This plasma direct exsolution method offers great potential to the practical deployment of exsolution‐based stable catalyst systems.

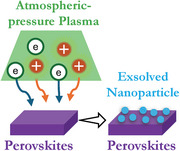

## 1 Introduction

Exsolution has demonstrated exceptional opportunities to deliver well‐dispersed nanoparticles (NPs) on oxide supports with unprecedented chemical and mechanical stability. Perovskite oxides, with a unique crystal structure (ABO_3_), are highly suitable supports to promote exsolution where the metal element at the B‐site for instance can exsolve out of the bulk and form uniformly distributed and socketed NPs at the surface. The driving force in exsolution of NPs is generally considered to be the lattice‐reduction which is further controlled or facilitated by defects (e.g., oxygen vacancies) and external conditions (atmosphere, temperature, strain etc.).^[^ ^1^, ^2^, ^3^, ^4^, ^5^, ^6^ ^]^ While exsolution from stoichiometric oxides has been demonstrated,^[^ ^7^ ^]^ non‐stoichiometry in perovskite oxides has allowed broadening the opportunities of exsolution and exsolved NPs.^[^ ^6^, ^8^ ^]^ For instance an A‐site deficient perovskite (A/B < 1) favors the exsolution of the element at the B‐site. Exsolved NPs have therefore generated great interest for energy applications where “resilient” NPs are often required.^[^ ^1^, ^2^, ^3^, ^4^, ^5^, ^9^, ^10^ ^]^ For instance NPs on the surface of an oxide can be effectively used in electrodes materials for solid oxide fuel cells and can act as active sites for catalytic reactions with enhanced activity.^[^ ^1^, ^4^, ^5^, ^10^, ^11^, ^12^, ^13^ ^]^

Exsolution has been initially achieved through high temperature (>300 °C) and long reducing treatments (e.g., with hydrogen for many hours).^[^ ^2^, ^3^, ^4^, ^14^, ^15^ ^]^ The use of various chemical compositions, strain, doping, metal‐organic frameworks, phase transition and defect strategies have also allowed exsolution at relatively low temperatures, without reducing gases and on binary oxides.^[^ ^6^, ^16^, ^17^, ^18^, ^19^, ^20^ ^]^ A range of other exsolution techniques are now being explored that are both contributing to the understanding of the common fundamental exsolution mechanisms as well as to expanding the application opportunities.^[^ ^21^, ^22^, ^23^ ^]^ High temperature but fast exsolution has been for instance demonstrated through an applied electrical potential (50% H_2_O/N_2_, <150 s).^[^ ^1^, ^24^ ^]^ A drastic reduction in the thermal energy required for exsolution has been achieved through intense pulse light (in ambient air, <20 ms) or through photo‐illumination with the assistance of trialkyl amine (hole donor).^[^ ^21^, ^25^ ^]^ Recently, the use of plasmas has been proposed to aid exsolution and for instance a low‐pressure plasma has been used in high temperature exsolution (>650 °C), which drastically reduced the treatment time (≈1 h) and offered the possibility of using different process gases (e.g., N_2_, Ar).^[^ ^26^ ^]^ However, we recently demonstrated the possibility of low‐pressure plasma direct exsolution at room temperature with an argon gas feed,^[^ ^27^ ^]^ which provided insights into the role of plasma species, surface defects and surface charging.

Plasmas are particularly suited for exsolution as they can produce surface oxygen defects through physical means (i.e., gas‐phase ion bombardment), drive the transport of the metal bulk oxide ions to the surface and contribute to adatom surface mobility to complete exsolution with NPs formation.^[^ ^27^ ^]^ A plasma exsolution process therefore requires neither thermal energy nor a reducing atmosphere, providing great opportunities to work with a wide range of supports and promote “chemistry at a point.”^[^ ^28^ ^]^ Further exceptional benefits such as costs reduction, manufacturing scalability and processing times could be achieved if we could operate plasma exsolution at atmospheric pressure. While low‐temperature low‐pressure plasma processing has been well known for many decades as a cornerstone of the semiconductor industry, the use of atmospheric pressure plasmas, in particular for materials processing, has surged only in more recent times following substantial advances in understanding plasma phenomena at atmospheric pressure and low‐temperature.^[^ ^29^, ^30^, ^31^, ^32^ ^]^

Here we have employed a plasma‐based approach known as dielectric barrier discharge (DBD), which operates both at atmospheric pressures and room temperature. This represents a significant progress with respect to the current state of research as it marks the first‐ever NP exsolution at those conditions. In particular all the following barriers have been lifted simultaneously: i) no requirement for a vacuum process (e.g., for plasmas) or any form of processing chamber (e.g., for thermochemical reduction), ii) no need for any form of heating required, iii) very short time for exsolution and iv) if required, no need for chemically reducing environments. These breakthroughs are opening the way for large scale and manufacturing exsolution processes, including in‐situ exsolution while extending exsolution to a much wider range of materials. We therefore demonstrate, for the first time, room‐temperature rapid (up to 15 min) exsolution by an atmospheric pressure plasma in a DBD configuration, where exsolution is initiated through physical means (i.e., through ion flux). This type of atmospheric pressure plasma configuration is also characterized by very low costs (capital investment and maintenance), particularly suitable to meet high volume manufacturing requirements and with a high degree of versatility (e.g., for in situ exsolution). We verify that exsolution can be achieved successfully with an inert gas (He) on La_0.43_Ca_0.37_Ni_0.06_Ti_0.94_O_2.955_ (LCTN) samples and subsequently investigate the impact of using He/H_2_ gas mixtures. Finally, we investigate the functionality of our exsolved Ni NPs with catalytic testing, which demonstrated much higher activity than the impregnated reference catalysts. We support our results with an in‐depth characterization at different stages of the DBD process that corroborates our description of the exsolution mechanisms at these conditions. In our analysis we also introduce a new approach to assessing the exsolved NP, through magnetic measurements. Finally, as not reported before we address for the first time the impact of hydrogen in a plasma‐based exsolution process.

## 2 Experimental Section

### 2.1 Preparation of Perovskite Oxides

A‐site deficient La_0.43_Ca_0.37_Ti_0.94_Ni_0.06_O_2.955_ (LCTN) oxide having a perovskite structure was synthesized by a standard solid‐state synthesis, using La_2_O_3_ (Pi‐Kem), CaCO_3_, TiO_2_ (Thermo Scientific Chemicals), and Ni(NO_3_)_2_ *x*6H_2_O (Across Organics) as raw materials. The La, Ca, and Ti precursor powders were thermally treated in air at 300 °C, with the exception of La_2_O_3_, treated at 800 °C instead, prior mixing in stoichiometric amounts, while warm. The precursors mixture and Ni nitrate were mixed using acetone and an ultrasonic probe (Hielscher UP200S). The dried mixture was calcined at 900 °C for 12 h and the resultant mixture was redispersed in acetone assisted by the ultrasonic probe, then dried. Pellets of 11 mm in diameter were then pressed from the powder mixture and they were sintered at 1400 °C for 12 h in air, with a ramp rate of 5 °C min^−1^, to form the perovskite phase. Unless otherwise stated, pellets were broken into four equal parts which were then used as separate samples to study the exsolution under an atmospheric pressure plasma process with a dielectric barrier configuration, hereafter a dielectric barrier discharge (DBD).

An undoped La_0.4_Ca_0.4_TiO_3_ (LCT) perovskite powder was also prepared with a modified solid‐state method to serve as a non‐exsolved reference material where the Ni metallic phase was deposited via wet impregnation. The LCT powder was used as support. Metal loading was selected based on the initial doping content of Ni in the perovskite samples (2 wt%). The perovskite support was dispersed in a dilute aqueous solution of Ni(NO_3_)_2_ *×*6H_2_O and the suspension remained under stirring for one hour, followed by water evaporation and drying at ≈90 °C overnight. The dried materials were calcined in air at 500 °C for 4 h. This final temperature was reached with a step of 10 °C min^−1^. The phase was obtained by reducing the materials under 5% H_2_/He at 900 °C for 4 h.

### 2.2 Plasma Treatment of Perovskites Oxides

**Figure 1:**
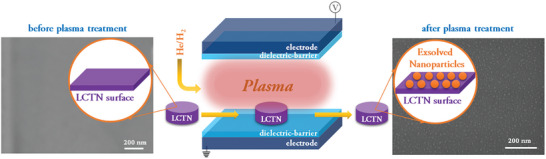
A schematic diagram showing the dielectric barrier discharge reactor used to treat perovskites oxides (La_0.43_Ca_0.37_Ni_0.06_Ti_0.94_O_2.955_, LCTN) to exsolve nanoparticles from the surface; SEM images are of untreated and treated LCTN sample in a He/1% H_2_‐fed DBD plasma for 15 min showing clearly the exsolution of NPs.

The surface treatment of the perovskite oxide samples was carried out with a DBD (**Figure** **1**, see also Section SI‐1 of the Supporting Information).^[^ ^33^ ^]^ Samples were treated as received to allow for comparison with other exsolution methodologies reported in the literature. The plasma was generated by applying a sinusoidal high voltage between two parallel plate electrodes (50 mm x 50 mm electrode area, 4 mm gas gap), both covered with a dielectric alumina plate.^[^ ^33^ ^]^ Plasma processes were carried out at a fixed excitation frequency of 20 kHz and an applied root mean square voltage of 1.2 kV. The applied voltage and current flowing through the circuit were measured with oscilloscope probes. The DBD electrode system was located into an airtight Plexiglas chamber, kept at constant pressure (10^5^ Pa) through gentle pumping with a diaphragm pump. Before DBD ignition, the chamber was purged with 8 standard litres per minute (sLm) of He for 15 min to reduce air contaminations. During the plasma processes, the samples were placed in the middle of the discharge region, onto the alumina plate covering the lower electrode. DBD plasmas fed with He and He/H_2_ mixtures were utilized. Helium flow rate was kept fixed at 6 sLm, while the H_2_ concentration [H_2_] in the He/H_2_ mixture was set at 0%, 0.6% and 1%. Process duration was optimized at 10 min. The power dissipated by the plasma was calculated as the integral over one cycle of the product of the applied voltage and the current and divided by the period;^[^ ^33^, ^34^ ^]^ this resulted to be about 9.3 ± 0.4 W and 10.5 ± 0.5 W when helium only or helium/hydrogen mixtures (any concentration) were used, respectively. The temperature was measured within the DBD plasma and found that under even the most exacting conditions, the electrode never reaches a temperature above 50 °C and under most conditions remains much lower, i.e., at room temperature. The measurement was carried out by placing a reversible temperature measuring strip in contact with the Ag/Pd electrode realized on the dielectric (alumina) plate. Furthermore, the electrical excitation conditions used in He DBD fall within the operational window of a homogeneous DBD in He (i.e., DBD in glow or diffuse regime).^[^ ^35^, ^36^ ^]^ While in the He/H_2_ fed DBD, under our selected conditions, the DBD operates in the filamentary regime.^[^ ^35^, ^36^ ^]^

### 2.3 Characterization of Perovskites Oxides

Samples were analyzed without any further treatment unless otherwise stated. The surface morphology of LCTN samples before and after DBD treatment were analysed using a Zeiss Supra 40 field‐emission scanning electron microscope (FE‐SEM) with an accelerating voltage of 2 kV. The size distribution of the exsolved NPs was produced using ImageJ software. Particle population densities were determined by taking the average of at least 4 images/areas. X‐ray photoelectron spectroscopy (XPS) was used to analyse the chemical composition of LCTN samples before and after the plasma treatment. A PHI P5000 VersaProbe II scanning XPS microprobe spectrometer equipped with a monochromatized Al Kα X‐ray source operated at a spot size of 100 µm (power of 14.8 W) was used. The pass energy and energy step size were respectively 117.4 and 1 eV for survey spectra, while for the high resolution spectra they were, respectively, 23.5 and 0.1 eV. Surface charging was compensated using a dual‐beam charge neutralization and spectra were calibrated with C 1s peak at 284.8 eV. The percent atomic concentrations were calculated from the high‐resolution spectra using the Scofield sensitivity factors set in the MultiPak software and a non‐linear Shirley background subtraction algorithm. The best‐fitting of the high‐resolution spectra was performed using the Multipak software. TEM analysis was carried out using a 2100F JEOL microscope with an operating voltage of 200 kV. The samples for TEM analysis were prepared by drop casting few drops of ethanol with a pipette, which were taken by gently scratching the surface of the pellets, onto the TEM grids. This approach produces a powder sample from the surface, however with limited control over the depth of sampling. The TEM grids were then allowed to dry in ambient conditions. High‐resolution TEM, selected area diffraction (SAED) patterns were acquired, and the lattice fringes (interplanar spacing) of the perovskite oxides and nanoparticles were analysed in Gatan Digital Micrograph. As the sample preparation can impact the morphology, surface area and crystal arrangements at the side of the pellets, the analysis of the nanoparticles was carried out considering only the pellets top surface for consistency and to allow a reliable comparison from sample to sample; consistent exsolution on the sides of the pellets was not observed.

### 2.4 Magnetic Measurements

Magnetic measurements were made on samples following plasma treatment using a Quantum Design MPMS3 operating in dc mode. All samples were mounted on the flat surface of a high purity quartz rod using a thin layer of vacuum grease. Samples were measured either as pieces of pellet, adhered to the rod on the underside of the pellet, or as thin layers on the surface of a layer of adhesive tape following exfoliation. The latter was carried out in order to greatly improve the visibility of the nanoparticle magnetic fraction above the ionic Ni^2+^ signal from the perovskite host. Control and background experiments on tape and on material exfoliated from the lower pellet surface were also undertaken.

### 2.5 Catalytic Testing

For catalytic testing, full pellets of 11 mm diameter (i.e., these were not broken down in four parts) were used to ensure a more rigorous and reliable catalytic characterization. The weight of the pellets was approximately 0.55 g. These were treated with the DBD plasma fed with He/H_2_ mixtures ([H_2_] = 0–1%) for 15 min. The particle size and population densities are shown in the Section SI‐2 (Supporting Information), (Figures S9–S14, Supporting Information), and Section SI‐4 (Supporting Information). A continuous flow single chamber reactor was used for the catalytic activity testing of the pellets. A k‐type thermocouple was placed in the proximity of the pellet catalyst samples and was used to measure the temperature during the experiment. All the experiments were carried out at atmospheric pressure. A fixed‐bed reactor filled by Al_2_O_3_ powder was placed upstream to the single chamber reactor and was used to decompose any carbonyl species from the CO‐containing gas cylinder during the CO oxidation experiments. For the CO oxidation, CO hydrogenation and the CH_4_ oxidation experiments 20% CO/He, 20% O_2_/He, 5% H_2_/He and 5% CH_4_/He and CP grade He cylinders were used, all provided by BOC. The He gas cylinder served as the balance gas to achieve the desired concentrations. A flow rate of 1 × 10^−4^ mol s^−1^ (150 mL min^−1^) was used throughout the experiments, using mass flow controllers supplied by BROOKS Instruments. In order to study the effect of temperature, the LCTN pellet catalyst samples were heated in the following inlet gas mixtures: a) 1% of O_2_ and 0.6% of CO from 200 °C up to 510 °C for the CO oxidation experiment, b) 3% of H_2_ and 1% of CO from 200 °C to 500 °C for the methanation from synthesis gas experiment and c) 0.5% of O_2_ and 1% of CH_4_ from 500 °C to 800 °C for the methane oxidation experiment. The temperature was held during the heating of the sample after each step of 20 °C. The holding time varied based on the time the reaction rate becomes steady meaning the rate of production does not change by more than ±5% over 1 h. The reaction rates were measured under gradient less conditions with the reactor operated under conditions of differential conversion (i.e., conversions below 20%). The CO_2_ mole fraction in the product stream was analysed with an XTREAM‐CO_2_ analyser from Rosemount. The minimum measurable CO_2_ mole fraction was 1 ppm which corresponds to a minimum measurable CO_2_ production rate of 1 × 10^−10^ mol s^−1^. The flow rates were also measured at the outlet using a Varian digital flow meter (1000 series). Reaction rates ($r_{CO_{2}}$) in terms of CO_2_ production are calculated as shown in Equation 1:

(1) $$r_{CO_{2}}\;\left(\mathrm{mol}(CO_{2})\;s^{-1}\right)=y_{CO_{2}}\;\cdot \dot{n}$$

where ($y_{CO_{2}}$) is the measured CO_2_ mole fraction at the gas outlet (measured by the XTREAM‐CO_2_ analyzer) and *ṅ* is the molar flow.

The CO and CH_4_ mole fractions in the product stream were analyzed with a Hiden QGA (HAS‐301‐1291) mass spectrometer (at m/z = 28 (CO) and 15 (CH_4_)) through a heated capillary line. Equation 1 was used to calculate the corresponding reaction rates where the measured CO mole fraction (*y*_CO_) and CH_4_ mole fraction $(y_{CH_{4}}$) where used instead, respectively.

Nominal turnover frequency (nTOF) is calculated as the number of molecules reacted per second, per exposed metal atom site at the surface of particles. To calculate nominal turnover frequency (number of molecules reacted per second, per expose metal atom site as the surface of the particles, nTOFs) for the catalyst systems, Equation 2 is used:

(2) $$\mathrm{nTOF}\;\left(s^{-1}\right)=10^{-20}\cdot N_{A}\cdot r_{CO_{2}}\;\cdot a^{2}/\left(A_{e}\cdot A_{p}\cdot k\right)$$

where: $r_{CO_{2}}$: is the reaction rate (mol s^−1^); N_A_: is the Avogadro's number (mol^−1^); *A_e_*: is the surface area of the pellet decorated with particles per total pellet surface area (µm^2^ µm^−2^); *A_p_*: is the exposed particle area per total surface area (cm^2^); *a*: is the unit cell parameter of the crystal lattice of the particles; *k*: is the average number of metal sites per unit cell face (the faces were considered to be in a (100) termination, thus, for the NiO rock‐salt structure *k* = 1).

Note that the product (*A_e_* · *A*)/*a* ^2^ · *k* gives the corresponding number of active sites. Equation (2) was used to calculate the corresponding nTOF values for CO and CH_4_, where the measured CO reaction rates (*r*_CO_) and CH_4_ reaction rates $(r_{CH_{4}}$) where used instead, respectively.

For the catalytic experiments with the powders, similar conditions were used but a fix packed‐bed reactor was used instead. The catalyst bed was made by using a total weight of 5 mg of 2% Ni‐LCT powder. This was chosen as such to model the approximate amount of Ni in the newly prepared perovskite samples and still be within the accuracy of the scale when making the catalyst bed.

## 3 Results and Discussion

### 3.1 Materials Characterization before and after Plasma Exsolution

Figure 1 represents a schematic diagram depicting the DBD process and reactor with representative SEM images of the sample surface before and after plasma exsolution. The LCTN samples are placed in the plasma region where they are exposed to the plasma.

**Figure 2:**
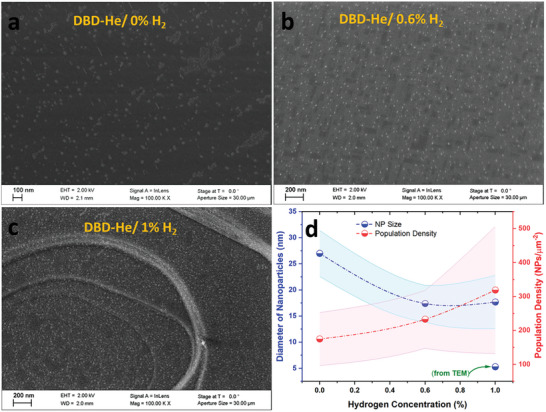
a–c) Field‐emission scanning secondary electron (topographic) micrographs of La_0.43_Ca_0.37_Ni_0.06_Ti_0.94_O_2.955_ (LCTN) treated in DBDs fed with He, He/0.6% H_2_, He/1% H_2_, respectively; d) a plot showing the changes in the diameter of DBD exsolved NPs and population densities with changing hydrogen concentrations from 0% to 1% (the shaded areas are drawn on the basis of the standard deviations).

**Figure** **2**a shows the SEM image of an LCTN sample treated in pure He plasma (0% H_2_) revealing the extent of exsolution of well‐dispersed NPs. Figure 2b,c shows SEM images of LCTN samples treated under helium/hydrogen plasma with 0.6% H_2_ and 1% H_2_ respectively. The exsolved NPs are homogeneously distributed throughout the surface of the treated samples. A summary of the size distribution and population density deduced from analyzing various SEM images (Figures S2–S8 in the Supporting Information) is displayed in Figure 2d. However, we should stress that statistical analysis is impacted by the limits of the SEM resolution and that a number of small particles may have escaped from the statistics because of the difficulties in imaging very small particles (< 4 nm) on this perovskite oxide. These could be observed during TEM analysis (“from TEM” in Figure 2d, see also Figures S18 and S19, Supporting Information), however TEM does not provide the opportunity to carry out a meaningful macroscopic statistical evaluation. Hence, we expect the population densities reported in Figure 2d to represent a lower bound, while a higher bound for the diameters. We should also note that the size/distribution analysis does not distinguish particles of different phases, e.g., metallic Ni or oxidized. With this premise, the average diameter of NPs was found to be around 27 nm when exsolved without hydrogen while the size of NPs is reduced to around 18 nm when H_2_ was included (for both 0.6% and 1% H_2_). The population density was 175 µm^−2^ without hydrogen, which increased to 233 µm^−2^ and 319 µm^−2^ for 0.6% and 1% H_2_ respectively as shown in Figure 2d. We have observed consistent exsolution throughout the surface of all the samples we have analyzed (Figures S2–S14 in the Supporting Information). This is probably further facilitated by the non‐conductive nature of the samples, which prevent consecutive microdischarges to reform close to each other due to charge accumulation on the surface. However, on a scale larger than the micrometer, the population density varied quite dramatically depending on the areas on the sample (Figure S8b, Supporting Information), leading to relatively large standard deviations (Figure 2d) in particular when 1% hydrogen was used.

Ni exsolution by various methods has been tested on LCTN (same composition as our samples), which allows for a good comparison. The diameter of exsolved Ni particles has varied from 1 nm to 45 nm,^[^ ^1^, ^15^, ^26^, ^27^, ^37^, ^38^ ^]^ hence our findings here are generally consistent with the literature results. Exsolution without hydrogen produces relatively large particles, similarly to high‐temperature reduction^[^ ^39^ ^]^ while the introduction of hydrogen appears to produce smaller particles with diameters closer to low‐pressure plasma‐thermal exsolution^[^ ^26^ ^]^ or direct low‐pressure plasma exsolution.^[^ ^27^ ^]^ However, as many factors contribute to exsolution and each method present different conditions, it would be difficult to link directly the particle size to the exsolution method. As for particle densities from LCTN, these have shown a much wider variability with values ranging from 7 µm^−2^ to 900 µm^−2^.^[^ ^1^, ^15^, ^26^, ^27^, ^37^, ^38^ ^]^ The population densities reported by Myung *et. al* from the LCTN samples were around 400 µm^−2^ achieved through electrochemical switching (i.e., voltage‐reduction at high temperature), which was compared to the exsolution at high temperatures with hydrogen gas (≈90 µm^−2^).^[^ ^1^ ^]^ With thermal‐shock induced exsolution, a particle density of around 61 µm^−2^ can be achieved within few seconds; also in this case, the results were compared to conventional chemical gas induced exsolution at high temperatures resulting in very low population densities (≈7 µm^−2^).^[^ ^38^ ^]^ Exsolution of Ni nanoparticles at 10% H_2_/N_2_ (900 °C for 10 h) resulted in a much lower population densities (50–60 µm^−2^) with an average diameter of around 40 nm.^[^ ^15^ ^]^ Thermal exsolution assisted by plasma reported a maximum population density (≈900 µm^−2^) of Ni nanoparticles in Ar‐plasma with an average diameter of around 8 nm.^[^ ^26^ ^]^ The population density of nanoparticles was lower (≈500 µm^−2^) when N_2_ was used instead of Ar for the plasma gas.^[^ ^26^ ^]^ Recently, we demonstrated direct plasma exsolution at low temperature that resulted in the particle size of around 20 nm with a maximum population density of around 550 µm^−2^.^[^ ^27^ ^]^ While exsolution density produced with our He‐only DBD aligns with the low densities observed in the literature, the introduction of hydrogen has a considerable impact to improve the number densities and produce values comparable with the highest densities measured with LCTN.

**Figure 3:**
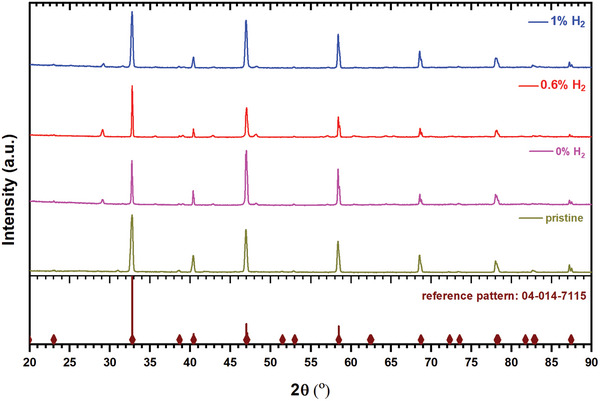
X‐ray diffraction of LCTN untreated and DBD treated samples. The peaks are well matched with perovskites oxides orthorhombic crystal structure.

**Figure** **3** shows the XRD spectra of LCTN samples before and after plasma treatments. The main peaks in the XRD are matching with the perovskite oxide crystal lattice (i.e., orthorhombic, reference pattern number: 04‐014‐7115). Ni peaks are not clearly visible from XRD (expected at 44.5°, 51.86°, 76.39°). Furthermore, the changes in the lattice parameters and cell volumes before and after plasma treatment can be seen in Section SI‐8 (Supporting Information). We observe some impurities in the pristine samples that vary from batch to batch that result in peaks such as those at ≈29°, ≈36°, ≈43° and ≈48°. However, these cannot be directly related to the plasma process as we observe them also in pristine samples and have no impact on the nanoparticles exsolution.

**Figure 4:**
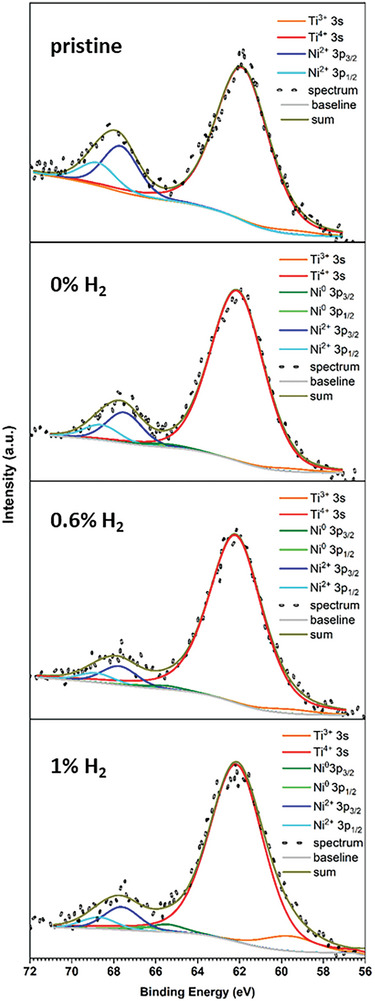
High resolution X‐ray photoelectron spectroscopy (XPS) of Ni 3p and Ti 3s core level regions revealing the appearance of metallic Ni (Ni^0^) after plasma treatment. The Ti^3+^ peak is also enhanced with plasma treatment.

XPS analysis has revealed the presence of metallic Ni in the high‐resolution spectra of Ni 3p in **Figure** **4** where the Ti 3s core level is also included. It can be seen that the metallic peak of Ni appears with the plasma treatment as shown in Figure 4 and summarized in **Table** **1**. In addition to Ni^0^, the peak of Ti^3+^ also increases, which suggests an increasing density in oxygen vacancies at the surface with increasing hydrogen concentration in the plasma feed mixture; this appears to be supported by the XPS analysis of the O 1s signal (Figure S17 in the Supporting Information), however we should note that the oxygen defect density at the surface is impacted by simultaneous processes (i.e., formation by ion bombardment, passivation by Ni ion reaching the surface etc., see further below). This is shown in Figure 4, corroborated by the analysis of the Ti 2p peak (Figure S16 in the Supporting Information) and summarized in Table 1.

**Table 1:** Fraction of metallic Ni and Ti^3+^ (as compared to the total Ni and total Ti, respectively), in pristine and plasma treated samples, as deduced from the deconvolution of the high‐resolution XPS spectra of Ni 3p, Ti 3s and Ti 2p. Full details of the positions of the curve‐fitting components of the Ni 3p, Ti 3s and Ti 2p signals that were used to obtain the results shown in this table are reported in Table S2, and Section SI‐3, Supporting Information).

Peak‐fit		Pristine	0% H_2_	0.6% H_2_	1% H_2_
Ni 3p	Ni^0^/(Ni^0^ + Ni^2+^)	0%	12%	18%	27%
Ti 2p	Ti^3+^/(Ti^3+^+ Ti^4+^)	1%	1%	2%	7%
Ti 3s	Ti^3+^/(Ti^3+^+ Ti^4+^)	2%	2%	2%	7%

According to XPS analysis, the fraction of Ni^0^ (expressed as percentage of the total Ni) in pristine LCTN, LCTN treated with pure He DBD, 0.6% H_2_ in He DBD and 1% H_2_ in He DBD are 0%, 12%, 18% and 27% respectively. Similarly, the Ti^3+^ fraction (expressed as percentage of the total Ti) is also increased in LCTN samples from 2% to 7% with increasing the concentration of hydrogen in the He DBD as summarized in Table 1. Hence, the presence of the Ni° component confirms the metallic nature of exsolved NPs while the Ni^2+^ comes mainly from the LCTN matrix which is consistent with the reported results from the literature.^[^ ^40^ ^]^ Furthermore, the XPS percent atomic concentrations of all the elements present in the LCTN samples before and after plasma treatments are summarized in Table S1 (Supporting Information).

**Figure 5:**
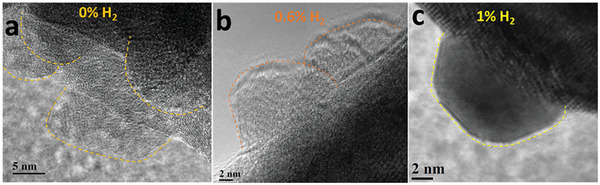
a–c) Representative high‐resolution TEM images of LCTN samples treated in He‐DBD with 0% H_2_, 0.6% H_2_ and 1% H_2_ respectively.

Exsolved nanoparticles were also examined under the TEM. **Figure** **5**a–c shows high‐resolution TEM images of exsolved NPs under the three different plasma treatment conditions. Our TEM analysis confirms typical characteristics observed after exsolution of nanoparticles, such as their socketed nature and crystallographic alignment with the hosting perovskite lattice. The individual nanoparticles are highlighted in each of the samples as shown in Figure 5a–c. The formation of the faceted nature of the exsolved NPs can be seen in all the samples but are more pronounced in the case of 1% H_2_ as shown in Figure 5c. However, we have been able to identify some differences due to the presence of hydrogen as a process gas. For this reason, we have carried out more in‐depth analyses comparing the samples treated with He‐only and those with 1% hydrogen (see Section 3.2).

In order to provide a more macroscopic assessment of the exsolved nanoparticles, we have explored the possibility of extracting useful information from magnetic measurements. Magnetic measurements were performed on samples following exsolution with He‐only or He/1% H_2_ DBD. Prior to exsolution the LCTN samples have a magnetic signature that is dominated by the Ni^2+^ ions within the perovskite host, though there is also a small diamagnetic contribution to the signal from the lattice. As the dilute Ni^2+^ ions are non‐interacting, the signal is very well described by a Brillouin function. At all but the lowest temperatures the measured field dependence of the magnetic moment μ can be modeled as μ  =  *HV* χ_p_, where *V* is the sample volume, *H* is the applied magnetic field and χ_p_ is the paramagnetic Curie susceptibility $\chi_{p}=\frac{C}{T}\;$. At room temperature, the Curie constant *C* is well described by the *S*  =  1 ground state for Ni^2+^. Following exsolution there are two main contributions to the signal from the sample. As only a small fraction of the Ni^2+^ ions in the sample exsolve to form nanoparticles on the upper pellet surface, the magnetic signal from Ni^2+^ ions remains as the dominant one (typically linear with field). The signal from the exsolved metallic nickel nanoparticles has the characteristic “S”‐shaped field dependence (Langevin function) of superparamagnetic particles. In actuality many of the particles are large enough to be fully `blocked’ at room temperature (not subject to superparamagnetic‐like fluctuations), so that the signature is essentially that of a very soft ferromagnet. In principle this is easily distinguished from the linear Ni^2+^ paramagnetic signal, except that the fraction from the nanoparticles is so small that this can be difficult to isolate with good precision (Section SI‐6, Supporting Information). Moreover, due to the Curie susceptibility of the paramagnetic signal, at low temperature the nanoparticle Ni° fraction is completely buried within the paramagnetic Ni^2+^ signal.

To produce clearer signatures of the nanoparticle metallic Ni^0^ signal, the upper layer of the LCTN pellet was exfoliated using clear adhesive tape, so that the adhered material gives a signal dominated by the nanoparticle fraction at the surface. This has the disadvantage that the amount of measured material cannot be quantified but carries the advantage that the signature of the nanoparticles is clearly resolved. The linear diamagnetic contribution to the signal from the quartz sample holder and tape are easily corrected for. Exfoliation from the underside of the samples was also carried out together with the corresponding measurements for comparison (Section SI‐6, Supporting Information).

**Figure 6:**
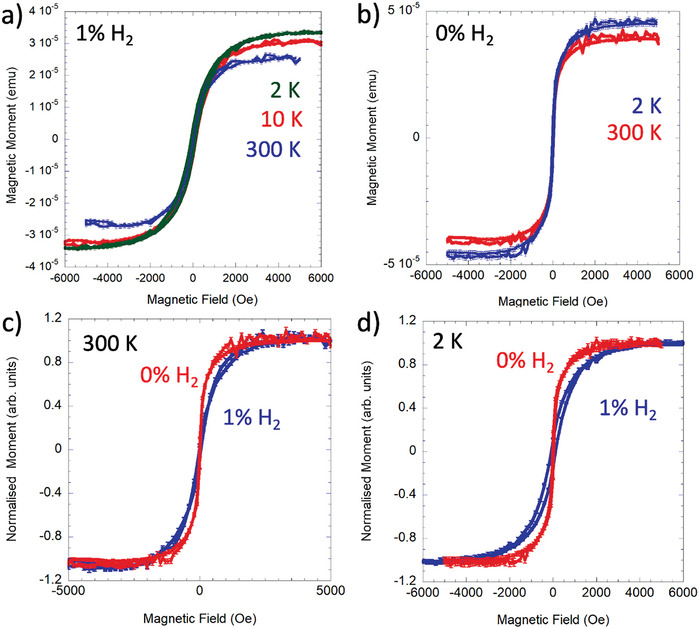
a) Magnetic moment as a function of applied field for an exfoliated sample after plasma treatment in He‐DBD with 1% H_2_. Measurements were made at temperatures of 300, 10, and 2 K (mass = 163.7 mg). b) Magnetic moment as a function of applied field for an exfoliated sample after plasma treatment in He‐DBD with 0% H_2_. Measurements were made at temperatures of 30 and 2 K (mass = 163.7 mg). c,d) Comparison of magnetic moment as a function of applied field for samples exfoliated from samples treated in He‐DBD with 0% H_2_ and 0% H_2_ The moments have been normalised to allow easy comparison. c) Measurements at 300 K. d) Measurements at 2 K. The paramagnetic signal dominates, which over this field range remains approximately linear.

**Figure** **6**a shows the signal for a sample of material exfoliated from the upper surface of a sample plasma treated using He‐DBD with 1% H_2_, which now effectively consists of signal originating entirely from the exsolved Ni particles that have adhered to the tape. The data have been corrected for a small residual diamagnetic contribution that derives from a combination of the adhesive tape and the quartz sample rod, which becomes noticeable due to the tiny amount of magnetic material that is now being measured. By contrast to measurements on the piece of broken pellet, cooling the exfoliated sample to 2 K reveals an enhanced ferromagnetic signal, as shown in Figure 6a. Interestingly there is even an increase in the saturated moment of around 33% between 300 and 2 K in this sample. For a given nanoparticle size the saturating field should capture the maximum magnetic signal even at 300 K (it is essentially described by a Langevin function for a superparamagnetic moment), which implies that at the lowest temperature of 2 K a larger number of nanoparticles are contributing to this component of the signal. This is consistent with a significant tail of much smaller nanoparticles that at high temperature effectively contribute to the paramagnetic background due to very fast superparamagnetic fluctuations of the smaller super‐moments. The size of this increase with temperature varies according to the exfoliated sample but is typically at least 10% of the 300 K moment. This variability requires further systematic investigation but is consistent with the variability and tails of size distributions observed via SEM measurements (Figure S8 in Supporting Information). There is also, as might be expected, an increase in the coercive width of the curves at low temperatures due to reduced thermal assistance of the magnetic reversal process.

Measurements were also made on a sample subject to He‐DBD with 0% H_2_, shown in Figure 6b. The results are generally similar to those measured on the 1% H_2_ sample, though some differences are worth highlighting. The increase in saturated moment on cooling from 300 to 2 K is less marked, suggesting a lesser influence from very small nanoparticles whose fluctuations freeze out at low temperature. This is reflected in the statistics from the SEM measurements (Figure 2d), where the average particle size tends to be larger for the He‐DBD 0% H_2_ samples. This is also evident in how the magnetization curves approach saturation, as illustrated in Figure 6c,d for measurements taken at both 300 and at 2 K. The He‐DBD 0% H_2_ reaches saturation with applied field more rapidly than the He‐DBD 1% H_2,_ which is consistent with larger average particle sizes in the former. The behavior is essentially described by the Langevin function, the sharpness of which depends upon the size of the particle moment (and hence on the particle volume assuming similar composition). For this particular sample the He‐DBD 1% H_2_ exhibits a greater hysteresis at low temperature, but the coercivity shows some variability across samples even when taken from the sample pellet, so more systematic work is required to comment on this further. While examining exfoliated samples reveals the presence, location (i.e., surfaces vs bulk) and many finer details of the nanoparticle behavior, this comes at the expense of a more quantitative analysis of overall nanoparticle yield and density. Future studies will pursue routes to a more quantitative analysis.

### 3.2 Discussion and In‐Depth Analysis on the Impact of Hydrogen

**Figure 7:**
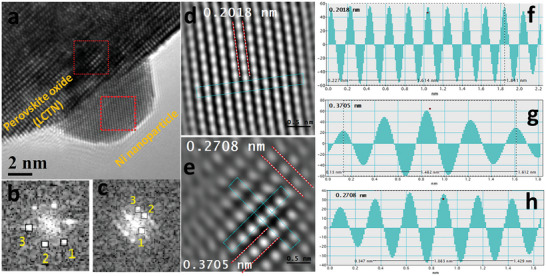
a) High resolution transmission electron microscopy (HR‐TEM) image of LCTN sample showing the morphology and faceted nature of exsolved Ni nanoparticle (NP), b,c) Fast Fourier transform (FFT) images taken from the highlighted red boxes on the Ni NP and LCTN lattice respectively. The spot numbers in (b) represent the Ni planes with spacing of 0.1772, 0.2042, 0.2014 nm, respectively. Similarly, the spot numbers in (c) correspond to the planes in LCTN with lattices spacing of 0.3739, 0.2746, and 0.2329 nm, respectively. d,e) The inverse FFTs of (c) and (d) clearly showing the lattices fringes of Ni and LCTN. f) The line profiles of the lattice fringes taken from the selected area in (d). f,g) The line profiles taken from the elected areas in (e). The average lattices fringes from the line profiles (as shown) are very close to the one taken from FFTs.

The samples exsolved without hydrogen addition in the DBD feed mixture are characterized by larger NPs with a lower density compared to the NPs produced with 0.6% and 1% H_2_ (Figure 2d). The metallic phase of Ni is also in general lower when hydrogen was not used compared to plasma treatment with H_2_ (Table 1). In our TEM analysis, we have been able to observe metallic Ni NPs with relative ease for the sample treated with 1% H_2_. For example, **Figure** **7**a shows the HR‐TEM of a 1% H_2_ LCTN sample. The exsolution of the NPs from the LCTN with a strongly faceted nature can be clearly seen, similar to previous reports.^[^ ^26^, ^37^ ^]^ The lattice fringes of exsolved NPs resembles very closely the planes of Ni NPs. The detailed HR‐TEM analysis of both the LCTN and Ni NPs lattices were performed by taking FFTs and line profiles from the lattice fringes of inverse‐FFTs as shown in Figure 7b–h. The FFTs spots in Figure 7b corresponds to the (200) plane (spot 1) and (111) planes (spot 2‐3) of Ni NPs. Similarly, the FFTs spots in Figure 7c corresponds to lattices spacing of 0.3739, 0.2746, and 0.2329 nm matching closely with the lattice planes of orthorhombic perovskite crystal. The lattice spacing obtained by taking the average value of the line profiles, in Figure 7f–h, were generated from the corresponding inverse‐FFTs as shown in Figure 7d,e. The average lattice spacing for the Ni NPs was found to be around 0.2018 nm (Figure 7d,f) matching with the standard (111) planes of Ni (0.2024 nm). Similarly, the lattice spacings for the LCTN resulted from line profiles are around 0.3705 nm and 0.2708 nm corresponding to the lattice planes of orthorhombic crystal system in perovskites as shown in Figure 7e,g,h. The exsolved NPs can be in the form of faceted or elliptical shapes as can be seen from the other TEM images shown in Figure S18 (Supporting Information).

**Figure 8:**
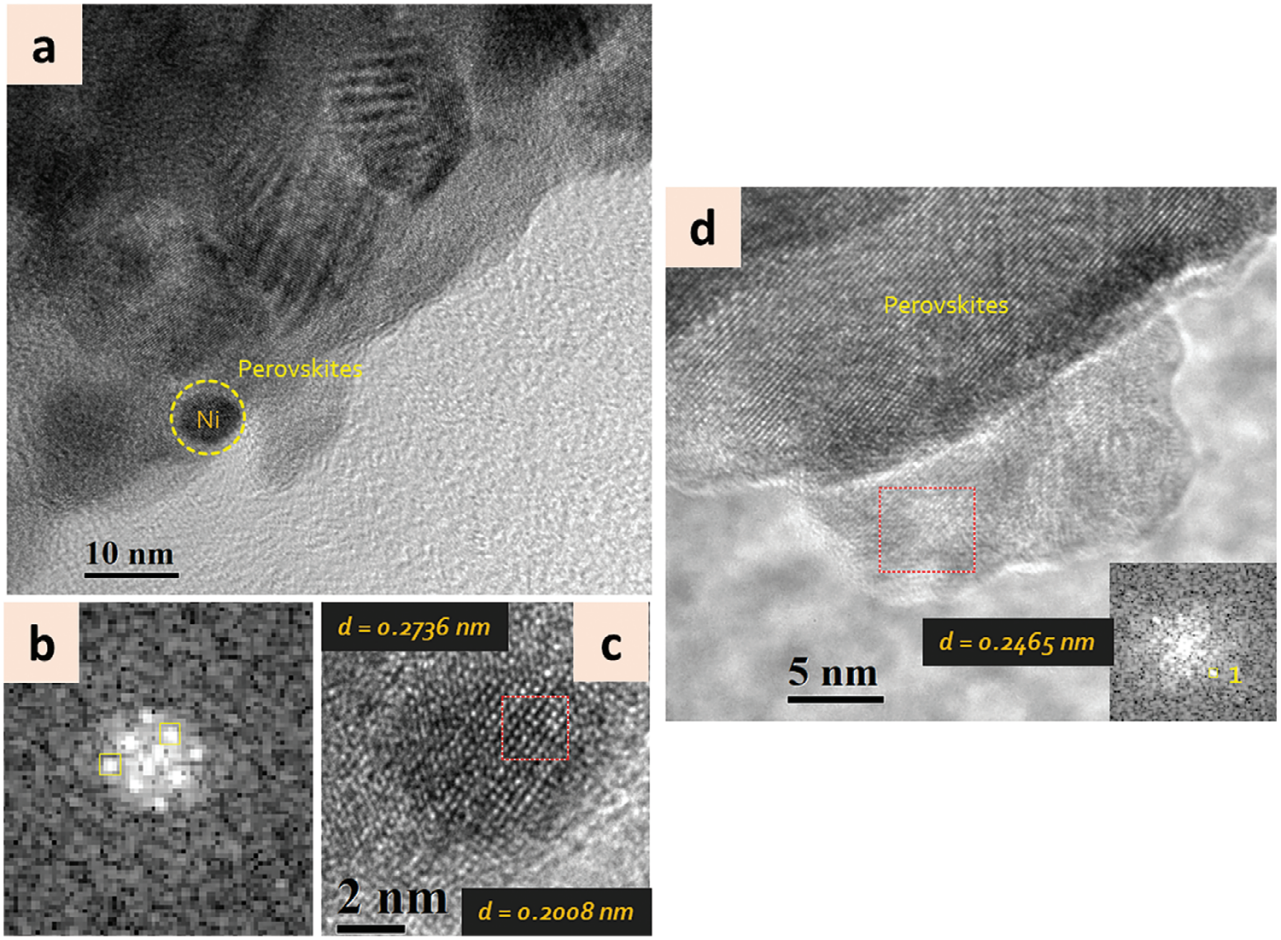
a) Transmission electron microscopy (TEM) of exsolved Ni NPs on the LCTN surface, treated in 0% H2 DBD. b) FFT of the highlighted region of the NPs shown in (c). c) Lattice fringes of the NP embedded in perovskite oxide taken from the highlighted region in (a) showing lattice spacing corresponding to both the perovskites oxide and metallic Ni. d) HR‐TEM representing larger NP exsolved from the perovskite matrix representing fringes closely matching with NiO.

The analysis of samples treated with He‐only plasma presented slightly different features, whereby together with metallic Ni NPs (**Figure** **8**a–c), we observed also oxidized NPs (Figure 8d) more often than for samples treated with hydrogen‐containing plasma. Figure 8c represents TEM images for 0% H_2_ treated LCTN samples showing the metallic nature of small Ni NPs. Larger NPs (around 20 nm) were also observed, which resulted to be oxidized to NiO as can be seen in Figure 8d, suggesting that the larger size may be due in part to oxidation.

These results indicate that a higher degree of process control is required through further understanding and optimization of plasma exsolution. However they also show that, within 10 minutes, NPs exsolution on the LCTN surface can be seen both in He‐DBD and He/H_2_‐DBD, demonstrating the potential of a low‐cost, fast and scalable atmospheric pressure exsolution process based on a DBD plasma, which is also particularly suitable to “promote chemistry at a point.”^[^ ^41^ ^]^

The fundamental mechanisms leading to exsolution in this atmospheric pressure plasma treatment is initiated by ion bombardment, which is responsible for the formation of oxygen vacancies and defects and creates the thermodynamic conditions for fast and low‐temperature exsolution.^[^ ^27^ ^]^ Surface charging also plays a key role in neutralizing Ni ions reaching the surface and enhancing surface diffusion.^[^ ^27^, ^42^, ^43^ ^]^ Overall, plasma exsolution is a surface process where endo‐particles have not been observed, hence differentiating in this respect from thermal exsolution where exsolved NPs have been observed within the bulk of the perovskite oxide.^[^ ^44^, ^45^ ^]^ Also, the existence of bulk particles is not only based on reduction conditions (i.e., plasma, hydrogen, bias) but also on material design.^[^ ^45^, ^46^, ^47^, ^48^ ^]^ One of these design principles is the doping level of the exsolvable ions, which in our case, is not such to allow for bulk exsolution. Having said that, even if bulk particles were present, they could not be responsible for the activity demonstrated, since there is a requirement of a driving force to access their capabilities which is not present in traditional catalytic processes (like the ones demonstrated here).

While exhibiting considerable similarities, there are however a number of important differences between low‐pressure and atmospheric pressure plasmas and between DBDs operated in the filamentary regime (e.g., with H_2_) compared to homogenous glow‐like or diffuse regime operation (e.g., He‐only). The filamentary regime also brings about spatial inhomogeneities at the surface on the µm to nm scale. Finally, the introduction of hydrogen also plays a role in differentiating these plasma processes. We believe that these differences are likely responsible for the different exsolution features observed, namely the creation of surface defects which vary in terms of both surface defect density as well as their nature (e.g., vacancies, dislocations with different energies etc.) and these have determined the NP size distribution, density and chemical composition/oxidation state.

In atmospheric pressure plasmas, the ion energy distribution supplies ions to a surface with energies that can reach high values.^[^ ^49^, ^50^, ^51^, ^52^, ^53^ ^]^ For instance a detailed simulation study on filamentary regimes (corresponding to our He‐H_2_ conditions), has shown that ions can reach energies above 100 eV for plasmas in contact with high relative permittivity (*ε*_r_ = 16) dielectric substrates (*ε*_r_ = 18 for our LCTN).^[^ ^27^, ^49^ ^]^ While these very high energy values may be representative of extreme conditions, the energy distribution also shows that a large fraction of ions have energies above that required to produce defects at the LCTN surface (> ≈6 eV).^[^ ^27^, ^49^ ^]^ In order to produce sufficient defect densities to promote exsolution at the LCTN surface, the ion flux should also be sufficiently high. At atmospheric pressure conditions and with plasma densities typical of DBD operation in the diffuse regime, the ion flux to the substrate (He‐only DBD) is at least equal to or greater than that produced in low‐pressure plasmas.^[^ ^27^ ^]^ Hence, ion bombardment has the same features required to produce surface defects and initiate exsolution in a DBD glow regime, as found at low pressure. Ions also carry potential energy and their recombination at the surface can also release substantial energy to produce defects.^[^ ^35^ ^]^ When hydrogen is introduced, and filamentation is observed, ion bombardment becomes modulated in time and restricted spatially due to the transient and localized nature of the filaments. We should therefore expect a reduction of the average ion flux, while the ion energy distribution remains favourable to defect creation.^[^ ^49^ ^]^ Although full simulations to produce accurate values for ion fluxes and energy distributions would be required at our specific conditions, our semi‐quantitative analysis suggests more defects and more effective exsolution can be achieved under a He‐only plasma. In part this is verified when, assuming the exsolved nanoparticles are metallic Ni (Section SI‐5 in Supporting Information), the exsolution depth and number of exsolved Ni atoms are calculated and show higher values of exsolution depth when samples were treated with the He‐only plasma. Atmospheric pressure plasmas may also contain a significant concentration of excited neutral species, e.g., He metastable atoms with long lifetime due to forbidden radiative transitions and have energies of 19.8 eV and 20.6 eV.^[^ ^54^, ^55^, ^56^ ^]^ These can release substantial energy on impact with the surface and also promote the formation of defects.

Due to a range of mechanisms of defect formation, we should expect also that the nature of defects varies. In particular, ion bombardment also creates oxygen radicals at the surface and promote higher surface mobility, enhancing oxidation reactions of the growing Ni nuclei and nanoparticles. This could explain a possible more extensive presence of oxidized nanoparticles in the samples treated with He‐only plasma. The presence of hydrogen can limit the oxidation, either by removing oxygen radicals at the surface as well as by reducing oxide nanoparticles through heterogeneous processes, in which the reduction reactions occur at the interface between the plasma and the solid oxide.^[^ ^57^ ^]^ Reduction of metal oxides by hydrogen plasmas is known to take place, where both thermodynamic as well as kinetic conditions favour the reduction even at room temperature.^[^ ^49^, ^57^, ^58^ ^]^ Hydrogen plasma contain very active species such as H, H^+^ and H_2_^*^ which show highly favourable reduction power.^[^ ^58^ ^]^

The role of hydrogen is therefore twofold as it impacts the state and regime of the plasma as well as the chemistry at the surface of the sample. For this reason, the changes in particle density and size distribution (Figure 2d) cannot be directly attributed to the chemical role of hydrogen at the surface as changes in the He ion flux and He metastable densities can have a drastic impact in the defect formation and exsolution process. In order to discern the contribution of each phenomenon, a range of plasma diagnostics (and/or simulations) will be required to be implemented and for instance to determine the ion‐flux at the surface. Overall, we can state that exsolution in atmospheric pressure plasma is driven by similar principles and physical phenomena as in low‐pressure plasma, while the contribution of additional gas species (e.g., metastables) is also possible. The plasma regime has also an impact on the exsolution outcome due to differences in ion energy densities and fluxes and hydrogen can be used effectively to tune the chemistry of the exsolving particles. This suggests that by adjusting the plasma chemistry using other precursor gases, nanoparticles with different chemical compositions (e.g., sulphides, nitrides etc.) may be produced.

### 3.3 Catalytic Performance of LCTN Samples

To study the catalytic performance of the atmospheric pressure and low temperature plasma exsolved nanoparticles and compare their performance with their high‐temperature analogues we employed full dense LCTN pellets that had been treated with the He‐only DBD (LCTN‐He) or with the DBD with 1% H_2_ (LCTN‐1%/H_2_) and used them as model systems. We should emphasize that we carry out this work to show that plasma exsolved NPs are capable of a high standard of catalytic performance, on par with NPs exsolved with other approaches; we do not claim here that plasma exsolution leads to NPs with superior properties, rather that plasma exsolution offer new opportunities as an exsolution process.^[^ ^59^ ^]^

The application performance was investigated in a number of important environmental and energy applications: a) methanation from synthesis gas (mixture of CO and H_2_) which is a key reaction in the so‐called coal‐to‐SNG (synthetic natural gas) process,^[^ ^60^ ^]^ b) CO oxidation reaction which is applied in automotive exhaust pollution control and air purification^[^ ^12^ ^]^ and c) CH_4_ oxidation which is an emerging technology for treating residual emissions from natural gas power plants.^[^ ^61^ ^]^ Furthermore, to illustrate the practical value of our results, we synthesized an undoped LCT perovskite powder to serve as a reference of a non‐exsolved sample where Ni nanoparticles were deposited via impregnation and compared it against the LCNT‐He and LCNT‐1%/H_2_ catalysts.

**Figure 9:**
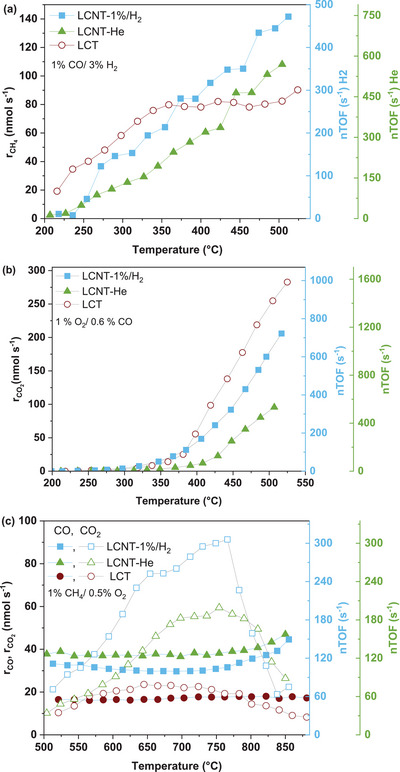
Catalytic activity high pressure‐low temperature plasma exsolved Ni nanoparticles using model (pellet) catalysts and of an undoped reference non‐exsolved catalyst. The reaction rates on the left y axis are not normalized. a) CH_4_ production rate from the CO methanation reaction, for all catalysts (left y axis) and nTOF values for the LCNT‐He and LCNT‐1%/H_2_ catalysts (right y axis) as a function of temperature, b) CO_2_ production rate from the CO oxidation reaction, for all catalysts (left y axis) and nTOF values for the LCNT‐He and LCNT‐1%/H_2_ catalysts (right y axis) as a function of temperature c) CO and CO_2_ production rates from the CH_4_ oxidation reaction for all catalysts (left y axis) and nTOF values for the LCNT‐He and LCNT‐1%/H_2_ catalysts (right y axis) as a function of temperature. The total gas flow rate for all experiments was 150 cm^3^ min^−1^.

**Figure** **9**a shows the CH_4_ production rate from the CO methanation reaction as a function of temperature (left y axis) for all catalysts and the corresponding nTOF values as a function of temperature (right y axis) for the LCNT‐He and LCNT‐1%/H_2_ catalysts. The nTOF values of the LCT catalyst were not possible to calculate as the sample was in a powder form which made the quantification of the surface nickel via SEM not possible. The rate on the left y axis is not normalized. The minimum temperature for any measurable CH_4_ reaction rate was approximately 300 °C for all catalysts (there was no measurable CO_2_ throughout the experiment). For all three catalysts, the rate of reaction increased upon increasing temperature. Across the entire examined temperature range, the CH_4_ production rate of the LCNT‐1%/H_2_ catalyst is higher than the corresponding rate of the LCNT‐He catalyst, due to the higher amount of total Ni metal exsolved (1.21 × 10^−3^ mg versus 1.11 × 10^−4^ mg, respectively). The non‐exsolved reference LCT catalyst showed slightly higher CH_4_ production rates up until approximately 350 °C compared to the exsolved catalysts but at higher temperatures that pattern was reversed. However, it should be noted that the LCT catalyst exhibits at least two orders of magnitude higher Ni content as opposed to the other two catalysts (1 × 10^−1^ mg as opposed to 1.11 × 10^−4^ mg and 1.21 × 10^−3^ mg of Ni exsolved on the surface of the pellets for the LCNT‐He and LCNT‐1%/H_2_ catalysts, respectively).

In order to better compare the site activities between the exsolved catalysts and to the literature data for base‐metal catalyst systems where the active phase is deposited via widely used top‐down techniques, we calculate their respective nTOF values for the CO methanation reaction. For this calculation, we assume that each surface metal atom serves as an active site for the methanation reaction and that the nanoparticles have hemispheric shape, consistent with analysis obtained from SEM images from Figure 2. For the exsolved LCNT‐He and LCNT‐1%/H_2_ catalysts the corresponding nTOF values are plotted against temperature on the right y‐axis in Figure 9a (green axis for LCNT‐He and blue axis for LCNT‐1%/H_2_; note the nTOF values for the LCT reference catalyst are not shown). The nTOF values for the LCNT‐He and LCNT‐1%/H_2_ plasma exsolved catalysts are of the order of 10^1^–10^2^ s^−1^. The nTOF values for the LCNT‐He catalyst are slightly higher than the LCNT‐1%/H_2_ catalyst across the entire examined temperature range. This difference largely originates from the fact that the particle population of the LCNT‐He sample is approximately 40% lower than that of the LCNT‐1%/H_2_ sample. Under similar reaction conditions, the nTOF values of the plasma exsolved catalysts are a few orders of magnitude higher than values reported in the literature for base metal oxide particles deposited using common top‐down deposition techniques, such as chemical impregnation.^[^ ^62^ ^]^

Figure 9b shows the CO_2_ production rate from the CO oxidation reaction as a function of temperature for all catalysts (left y axis) and the corresponding nTOF values as a function of temperature (right y axis) for the LCNT‐He and LCNT‐1%H_2_ catalysts. Similarly to the CO methanation reaction, for all three catalysts, the activity increased upon increasing temperature and the CO_2_ production rate of the LCNT‐1%/H_2_ catalyst was higher than the corresponding rate of the LCNT‐He catalyst. The catalytic performance of the reference LCT catalyst in terms of CO_2_ production rate was of the same order of magnitude as compared to the exsolved analogues even though its Ni content was at least two orders of magnitude higher. The nTOF values of the LCNT‐He and LCNT‐1%/H_2_ plasma exsolved catalysts are again of the order of 10^1^–10^2^ s^−1^ which is of the same order of magnitude as for thermally exsolved nanoparticles or similar nanoparticles exsolved by plasma at low pressure.^[^ ^27^, ^28^, ^63^ ^]^ For the CO oxidation reaction, the nTOF values of the LCNT‐1%/H_2_ catalyst are higher than the those of the LCNT‐He catalyst across the entire examined temperature range. This is probably due to the higher content of metallic Ni particles over the oxidized Ni particles observed in the LCNT‐H_2_ catalysts. Furthermore, the reported nTOF values of this study exceed the values reported in literature under similar experimental conditions for base metal oxide nanoparticles deposited using common deposition techniques (such as chemical impregnation) by a few orders of magnitudes.^[^ ^28^ ^]^

Figure 9c demonstrates the performance of the catalysts in terms of CO and CO_2_ production rates during the methane oxidation reaction for all catalysts and the corresponding nTOF values as a function of temperature (right y axis) for the LCNT‐He and LCNT‐1%H_2_ catalysts. The production rate of the LCNT‐1%/H_2_ catalyst was higher than the corresponding rate of the LCNT‐He catalyst and could also be attributed to the higher concentration of metallic Ni nanoparticles on the former as compared to the oxidized ones in the latter as well as the higher total amount of Ni exsolved. Production rates of CO were relatively similar. Overall both exsolved catalysts presented much higher activity than the impregnated reference catalyst across the entire temperature range. The catalytic performance of the reference LCT catalyst was of the same order of magnitude as compared to the exsolved analogues even though its Ni content is at least two orders of magnitude higher. This is likely due to the much larger size of the Ni particles (in the order of 70 nm) within the impregnated samples. It is also worth noting that plasma exsolved nanoparticles do not only demonstrate high catalytic performance as their thermally exsolved counterparts but are also capable of maintaining that performance during long‐term operation. To demonstrate that we show that the catalytic activity of the LCNT‐He catalyst towards the methane oxidation reaction at 800 °C is maintained for over 120 h of continuous testing, consistent with the well‐anchored nature of these particles, as opposed to the impregnated LCT catalyst where severe deactivation occurred within 1 h (Figure S25 in the Supporting Information).

## 4 Conclusion

This work has demonstrated for the first time how a DBD at atmospheric pressure can be used for exsolution of NPs from perovskite oxides at room temperature both with inert gases as well as with hydrogen‐containing mixtures. Similar to other recent developments in exsolution, DBDs resulted in a low‐temperature and very fast and higher growth rate of NPs than more conventional hydrogen thermochemical reduction. These exsolved NPs have also shown competitive performance results for various catalytic processes. DBD processing at atmospheric pressure also offer opportunities for scalability and a versatile approach for future manufacturing of exsolved NPs. In addition, the possibility of using a range of gas mixtures with highly reactive plasma species will allow exploring a very wide range of chemical reactions to transform their composition, structure and functionality.

## Conflict of Interest

The authors declare no conflict of interest.

## Supporting information

Supporting Information

## Data Availability

This paper is accompanied by representative samples of experimental data and the relevant numerical tabulated raw data is available from Ulster University’s Research Portal at https://doi.org/10.15129/e2e11901‐92c4‐4b2e‐a83e‐ff25052e972a. Detailed procedures explaining how these representative samples were selected, and how these experiments can be repeated, are provided in the corresponding sections of this paper. Additional results and raw data underlying this work are available in the Supporting Information or on request following instructions provided at https://doi.org/10.15129/e2e11901‐92c4‐4b2e‐a83e‐ff25052e972a.

## References

[1] J. H. Myung , D. Neagu , D. N. Miller , J. T. Irvine , Nature 2016, 537, 528. 27548878 10.1038/nature19090

[2] Y. Gao , D. Chen , M. Saccoccio , Z. Lu , F. Ciucci , Nano Energy 2016, 27, 499.

[3] J. T. S. Irvine , D. Neagu , M. C. Verbraeken , C. Chatzichristodoulou , C. Graves , M. B. Mogensen , Nat. Energy 2016, 1, 15014.

[4] Y. Li , W. Zhang , Y. Zheng , J. Chen , B. Yu , Y. Chen , M. Liu , Chem. Soc. Rev. 2017, 46, 6345. 28920603 10.1039/c7cs00120g

[5] K. Kousi , C. Tang , I. S. Metcalfe , D. Neagu , Small 2021, 17, 2006479. 10.1002/smll.202006479 33787009

[6] D. Neagu , G. Tsekouras , D. N. Miller , H. Ménard , J. T. S. Irvine , Nat. Chem. 2013, 5, 916. 24153368 10.1038/nchem.1773

[7] W. Kobsiriphat , B. D. Madsen , Y. Wang , M. Shah , L. D. Marks , S. A. Barnett , J. Electrochem. Soc. 2010, 157, B279.

[8] E. Y. Konysheva , X. Xu , J. T. S. Irvine , Adv. Mater. 2012, 24, 528. 22250062 10.1002/adma.201103352

[9] S. Liu , Q. Liu , J. L. Luo , ACS Catal. 2016, 6, 6219.

[10] B. Hua , M. Li , Y. F. Sun , J. H. Li , J. L. Luo , ChemSusChem 2017, 10, 3333. 28646521 10.1002/cssc.201700936

[11] O. Kwon , S. Sengodan , K. Kim , G. Kim , H. Y. Jeong , J. Shin , Y. W. Ju , J. W. Han , G. Kim , Nat. Commun. 2017, 8, 15967. 28656965 10.1038/ncomms15967 PMC5493762

[12] E. Calì , G. Kerherve , F. Naufal , K. Kousi , D. Neagu , E. I. Papaioannou , M. P. Thomas , B. S. Guiton , I. S. Metcalfe , J. T. S. Irvine , D. J. Payne , ACS Appl. Mater. Interfaces 2020, 12, 37444. 32698571 10.1021/acsami.0c08928

[13] Y. F. Sun , Y. Q. Zhang , J. Chen , J. H. Li , Y. T. Zhu , Y. M. Zeng , B. S. Amirkhiz , J. Li , B. Hua , J. L. Luo , Nano Lett. 2016, 16, 5303. 27455174 10.1021/acs.nanolett.6b02757

[14] Y. Zhu , J. Dai , W. Zhou , Y. Zhong , H. Wang , Z. Shao , J. Mater. Chem. A 2018, 6, 13582.

[15] V. Kyriakou , D. Neagu , E. I. Papaioannou , I. S. Metcalfe , M. C. M. van de Sanden , M. N. Tsampas , Appl. Catal., B 2019, 258, 117950.

[16] J. S. Jang , J. K. Kim , K. Kim , W. G. Jung , C. Lim , S. Kim , D. H. Kim , B. J. Kim , J. W. Han , W. C. Jung , I. D. Kim , Adv. Mater. 2020, 32, 2003983.

[17] J. Tan , D. Lee , J. Ahn , B. Kim , J. Kim , J. Moon , J. Mater. Chem. A 2018, 6, 18133.

[18] H. Han , J. Park , S. Y. Nam , K. J. Kim , G. M. Choi , S. S. P. Parkin , H. M. Jang , J. T. S. Irvine , Nat. Commun. 2019, 10, 1471. 30931928 10.1038/s41467-019-09395-4 PMC6443801

[19] S. Park , D. Oh , J. Ahn , J. K. Kim , D. Kim , S. Kim , C. Park , W. Jung , I. Kim , Adv. Mater. 2022, 34, 2201109. 10.1002/adma.202201109 35502659

[20] Y. Tian , C. Yang , Y. Wang , M. Xu , Y. Ling , J. Pu , F. Ciucci , J. T. S. Irvine , B. Chi , J. Mater. Chem. A 2022, 10, 16490.

[21] Z. Chen , B. Hua , X. Zhang , L. Chen , Y. Q. Zhang , G. Yang , G. Wan , H. Zhou , Y. Yang , J. Chen , H. Fan , Q. Li , M. Li , J. Li , W. Zhou , Z. Shao , J. L. Luo , Y. Sun , Cell Rep. 2020, 1, 100243.

[22] G. Weng , K. Ouyang , X. Lin , S. Wen , Y. Zhou , S. Lei , J. Xue , H. Wang , Adv. Funct. Mater. 2022, 32, 2205255.

[23] O. Kwon , S. Joo , S. Choi , S. Sengodan , G. Kim , JPhys Energy 2020, 2, 032001.

[24] W. Fan , B. Wang , R. Gao , G. Dimitrakopoulos , J. Wang , X. Xiao , L. Ma , K. Wu , B. Yildiz , J. Li , J. Am. Chem. Soc. 2022, 144, 7657. 35471024 10.1021/jacs.1c12970

[25] E. Shin , D.‐H. Kim , J.‐H. Cha , S. Yun , H. Shin , J. Ahn , J.‐S. Jang , J. W. Baek , C. Park , J. Ko , S. Park , S.‐Y. Choi , I.‐D. Kim , ACS Nano 2022, 16, 18133. 36108309 10.1021/acsnano.2c05128

[26] V. Kyriakou , R. K. Sharma , D. Neagu , F. Peeters , O. De Luca , P. Rudolf , A. Pandiyan , W. Yu , S. W. Cha , S. Welzel , M. C. M. van de Sanden , M. N. Tsampas , Small Methods 2021, 5, 2100868. 10.1002/smtd.202100868 34928018

[27] H. Khalid , A. ul Haq , B. Alessi , J. Wu , C. D. Savaniu , K. Kousi , I. S. Metcalfe , S. C. Parker , J. T. S. Irvine , P. Maguire , E. I. Papaioannou , D. Mariotti , Adv. Energy Mater. 2022, 12, 2201131.

[28] D. Neagu , E. I. Papaioannou , W. K. W. Ramli , D. N. Miller , B. J. Murdoch , H. Ménard , A. Umar , A. J. Barlow , P. J. Cumpson , J. T. S. Irvine , I. S. Metcalfe , Nat. Commun. 2017, 8, 1855 29187751 10.1038/s41467-017-01880-y PMC5707356

[29] D. Mariotti , T. Belmonte , J. Benedikt , T. Velusamy , G. Jain , V. Švrček , Plasma Processes Polym. 2016, 13, 70.

[30] U. R. Kortshagen , R. M. Sankaran , R. N. Pereira , S. L. Girshick , J. J. Wu , E. S. Aydil , Chem. Rev. 2016, 116, 11061. 27550744 10.1021/acs.chemrev.6b00039

[31] A. Uricchio , F. Fanelli , Processes 2021, 9, 2069.

[32] W. Chiang , D. Mariotti , R. M. Sankaran , J. G. Eden , K. (Ken) Ostrikov , Adv. Mater. 2020, 32, 1905508. 10.1002/adma.201905508 31854023

[33] F. Fanelli , F. Fracassi , A. Lapenna , V. Angarano , G. Palazzo , A. Mallardi , Adv. Mater. Interfaces 2018, 5, 1801373. 10.3390/ma13092181 PMC7254212 32397486

[34] F. Fanelli , F. Fracassi , Plasma Processes Polym. 2016, 13, 470.

[35] F. Massines , C. Sarra‐Bournet , F. Fanelli , N. Naudé , N. Gherardi , Plasma Processes Polym. 2012, 9, 1041.

[36] R. Brandenburg , Plasma Sources Sci. Technol. 2017, 26, 053001.

[37] D. Neagu , V. Kyriakou , I. L. Roiban , M. Aouine , C. Tang , A. Caravaca , K. Kousi , I. Schreur‐Piet , I. S. Metcalfe , P. Vernoux , M. C. M. Van De Sanden , M. N. I. Tsampas , ACS Nano 2019, 13, 12996. 31633907 10.1021/acsnano.9b05652

[38] Z. Sun , W. Fan , Y. Bai , Adv. Sci. 2022, 9, 2200250. 10.1002/advs.202200250 PMC9036016 35187861

[39] M. Chanthanumataporn , J. Hui , X. Yue , K. Kakinuma , J. T. S. Irvine , K. Hanamura , Electrochim. Acta 2019, 306, 159.

[40] S. Yu , D. Yoon , Y. Lee , H. Yoon , H. Han , N. Kim , C.‐J. J. Kim , K. Ihm , T.‐S. S. Oh , J. Son , Nano Lett. 2020, 20, 3538. 32271584 10.1021/acs.nanolett.0c00488

[41] D. Neagu , E. I. Papaioannou , W. K. W. Ramli , D. N. Miller , B. J. Murdoch , H. Ménard , A. Umar , A. J. Barlow , P. J. Cumpson , J. T. S. Irvine , I. S. Metcalfe , Nat. Commun. 2017, 8, 1855. 29187751 10.1038/s41467-017-01880-y PMC5707356

[42] I. Levchenko , K. Ostrikov , K. Diwan , K. Winkler , D. Mariotti , Appl. Phys. Lett. 2008, 93, 183102.

[43] H. Gu , W. Chen , X. Li , J. Mater. Chem. A 2022, 10, 22331.

[44] K. Syed , J. Wang , B. Yildiz , W. J. Bowman , Nanoscale 2022, 14, 663. 34874392 10.1039/d1nr06121f

[45] K. Kousi , D. Neagu , L. Bekris , E. I. Papaioannou , I. S. Metcalfe , Angew. Chem., Int. Ed. 2020, 59, 2510. 10.1002/anie.201915140 31804017

[46] K. Kousi , D. Neagu , L. Bekris , E. Calì , G. Kerherve , E. I. Papaioannou , D. J. Payne , I. S. Metcalfe , J. Mater. Chem. A 2020, 8, 12406.

[47] K. Kousi , D. Neagu , I. S. Metcalfe , Catalysts 2020, 10, 468.

[48] J. Wang , K. Syed , S. Ning , I. Waluyo , A. Hunt , E. J. Crumlin , A. K. Opitz , C. A. Ross , W. J. Bowman , B. Yildiz , Adv. Funct. Mater. 2022, 32, 2108005.

[49] N. Y. Babaeva , M. J. Kushner , Plasma Sources Sci. Technol. 2011, 20, 035017.

[50] N. Y. Babaeva , G. V. Naidis , Plasma Sources Sci. Technol. 2020, 29, 095020.

[51] N. Y. Babaeva , M. J. Kushner , Plasma Sources Sci. Technol. 2011, 20, 035018.

[52] J. Choi , F. Iza , J. K. Lee , C.‐M. Ryu , IEEE Trans. Plasma Sci. 2007, 35, 1274.

[53] N. Y. Babaeva , Plasma Processes Polym. 2017, 14, 1600165.

[54] K. Niemi , J. Waskoenig , N. Sadeghi , T. Gans , D. O'Connell , Plasma Sources Sci. Technol. 2011, 20, 055005.

[55] N. Miura , J. Hopwood , Rev. Sci. Instrum. 2009, 80, 113502. 19947726 10.1063/1.3258198

[56] I. Korolov , M. Leimkühler , M. Böke , Z. Donkó , V. Schulz‐von der Gathen , L. Bischoff , G. Hübner , P. Hartmann , T. Gans , Y. Liu , T. Mussenbrock , J. Schulze , J. Phys. D: Appl. Phys. 2020, 53, 185201.

[57] K. C. Sabat , A. B. Murphy , Metall. Mater. Trans. B 2017, 48, 1561.

[58] K. C. Sabat , Plasma Chem. Plasma Process. 2021, 41, 1329.

[59] Z. Sun , C. Hao , S. Toan , R. Zhang , H. Li , Y. Wu , H. Liu , Z. Sun , J. Mater. Chem. A 2023, 11, 17961.

[60] P. Panagiotopoulou , D. I. Kondarides , X. E. Verykios , Appl. Catal., A 2008, 344, 45.

[61] M. García‐Vázquez , P. Marín , S. Ordóñez , K. Li , J. Tan , G. Zhang , F. R. García‐García , J. Environ. Chem. Eng. 2021, 9, 106880.

[62] L. T. Thompson , J. Schwank , M. D. C. O. Curtis , AIChE J. 1989, 35, 109.

[63] E. I. Papaioannou , D. Neagu , W. K. W. Ramli , J. T. S. Irvine , I. S. Metcalfe , Top. Catal. 2019, 62, 1149.

