# Supplementary material for: Dielectric Barrier Plasma Discharge Exsolution of Nanoparticles at Room Temperature and Atmospheric Pressure
Source: Adv Sci (Weinh). 2024 Jul 4;11(34):2402235. doi: 10.1002/advs.202402235 (PMC11425884; doi:10.1002/advs.202402235)
Supplement: Supplementary file 1 — Supporting Information [file ADVS-11-2402235-s001.docx]

**Supplementary Information**

**Dielectric barrier plasma discharge exsolution of nanoparticle at room temperature and atmospheric pressure**

Atta ul Haq,^1#^ Fiorenza Fanelli,^2#^ Leonidas Bekris,^3^ Alex Martinez Martin,^3^ Steve Lee,^4^ Hessan Khalid,^1^ Cristian D. Savaniu,^5^ Kalliopi Kousi,^6^ Ian S. Metcalfe,^3^ John T. S. Irvine,^5^ Paul Maguire,^1^ Evangelos I. Papaioannou,^3^ Davide Mariotti^7^*

^1^School of Engineering, Ulster University, Belfast, BT37 0QB, UK

^2^Institute of Nanotechnology (NANOTEC), National Research Council (CNR), via Orabona 4, 70125 Bari, Italy

^3^School of Engineering, Newcastle University, Newcastle upon Tyne NE1 7RU, UK

^4^School of Physics and Astronomy, University of St. Andrews, Scotland Fife KY16 9SS, UK

^5^School of Chemistry, University of St. Andrews, Scotland Fife KY16 9ST, UK

^6^School of Chemistry & Chemical Engineering, University of Surrey, Guildford, Surrey, GU2 7XH, UK

^7^Department of Design, Manufacturing & Engineering Management, University of Strathclyde, Glasgow G1 1XJ, UK

*Corresponding author

^#^Equal contribution

**SI-1: Details about the plasma reactor**

The dielectric-barrier discharge (DBD) reactor as shown in Figure S1, also in Figure 1 of the main manuscript, consists of two parallel plate electrodes (50 mm x 50 mm) separated by a dielectric medium (alumina) with a gap of 4 mm. The samples are placed on top of the bottom electrode and are treated with a DBD plasma fed with He/H_2_ mixtures. The H_2_ concentration in the mixture was varied from 0-1%.

**
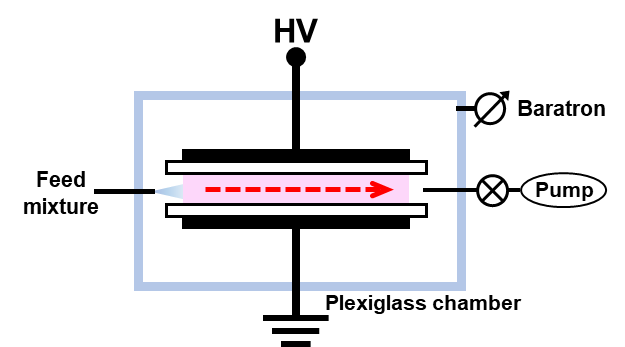
**

**Figure S1**: A schematic diagram showing the design and the main components of the dielectric barrier discharge reactor used to treat perovskites oxides (La_0.43_Ca_0.37_Ni_0.06_Ti_0.94_O_2.955_, LCTN) to exsolve nanoparticles from the surface.

**SI-2: Details about the size distributions of exsolved nanoparticles**

The size distribution and population densities of exsolved nanoparticles (NPs) were estimated using the ImageJ software. The details about the procedure of image processing in the ImageJ for size distribution is given elsewhere.^1^ The size distributions in ImageJ were carried out on regions of 4-6 SEM images which were then plotted in Origin software and displayed in Figure S2-7 for broken pellets and in Figure 9-13 for full pellets, the latter refers to catalytic testing. The summary of size distribution and population densities of NPs in broken and full pellets are shown as bar charts in Figure S8 and in Figure S14, respectively. The population density for each image is calculated dividing the counts by the area of the SEM image considered. An average value is calculated from all the images for size as well as for the population densities which are then shown in Figure 2d and in Figure S20 for broken and full pellets respectively.

**
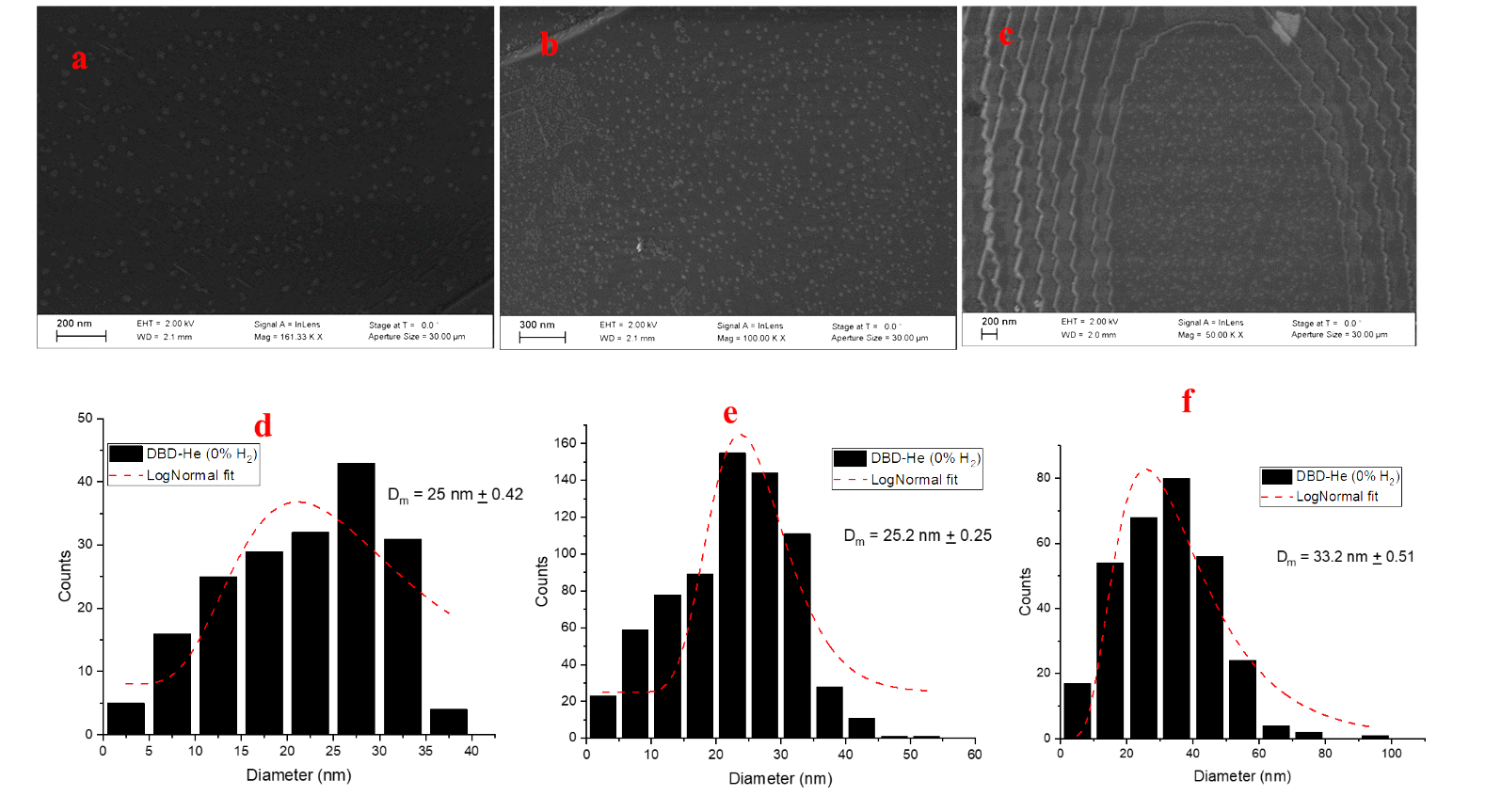
**

**
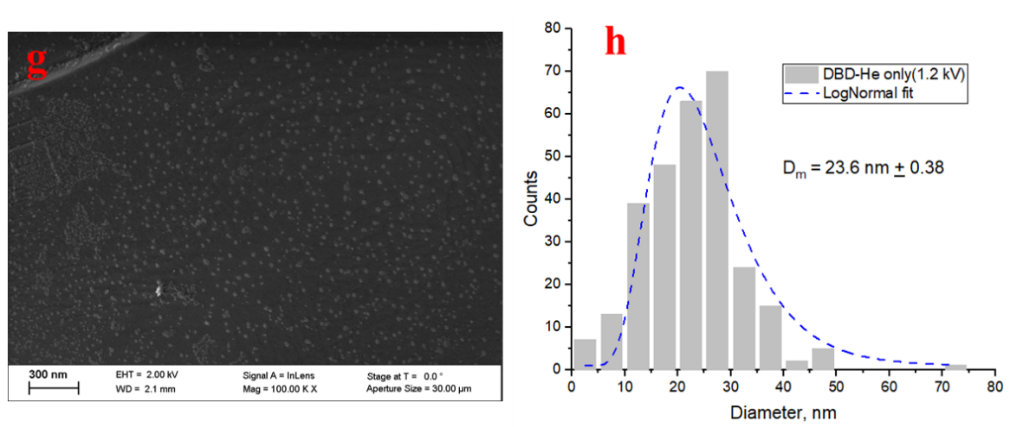
**

**Figure S2 (a-c, g)** FE-SEM images of **LCTN broken samples** treated in **He-DBD without hydrogen**; **(d-f, h)** the corresponding size distribution of exsolved NPs.


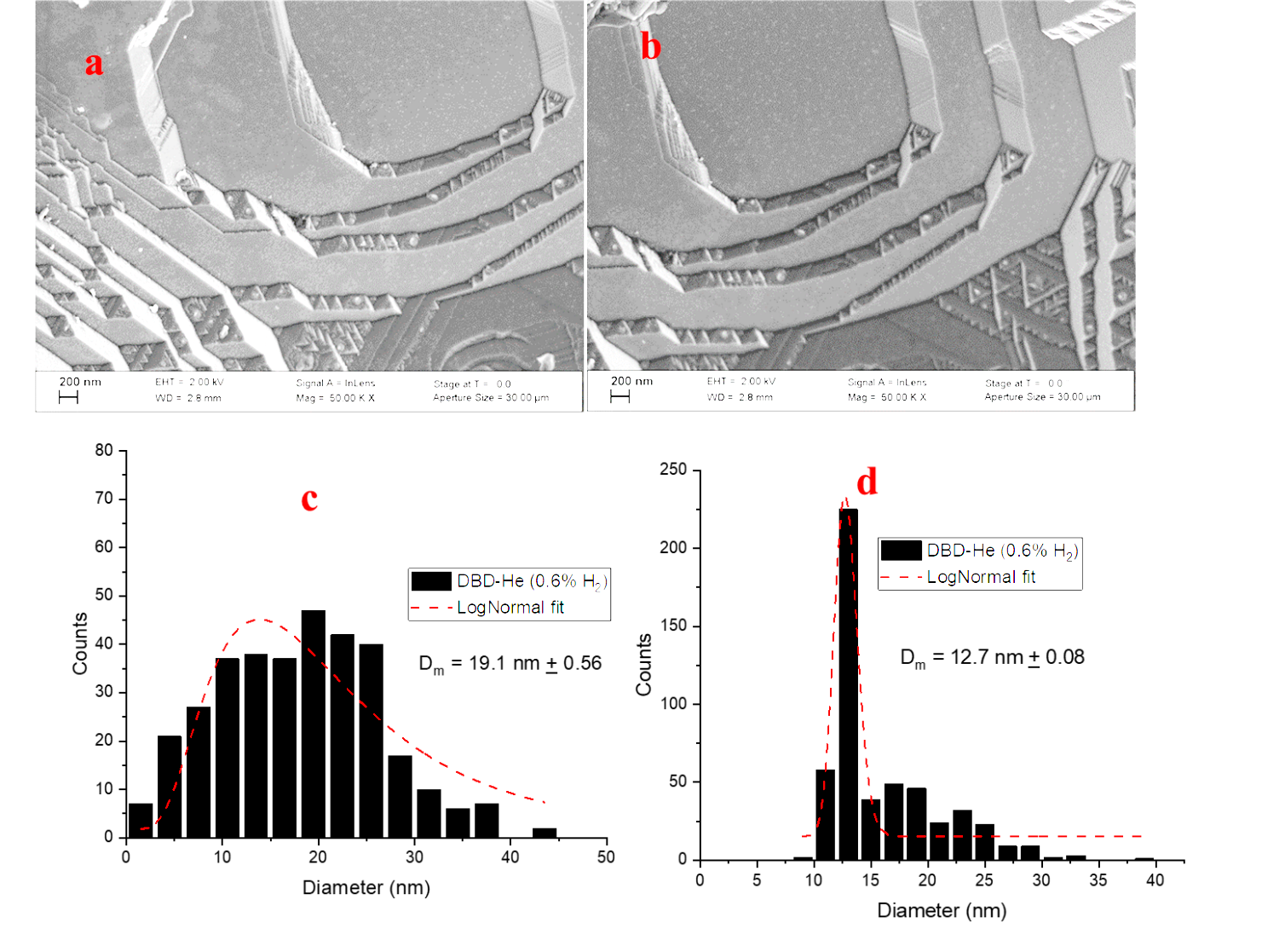


**Figure S3 (a-b)** FE-SEM images of **LCTN broken samples** treated in **He-DBD with 0.6 % hydrogen**; **(c-d)** the corresponding size distribution of exsolved NPs.


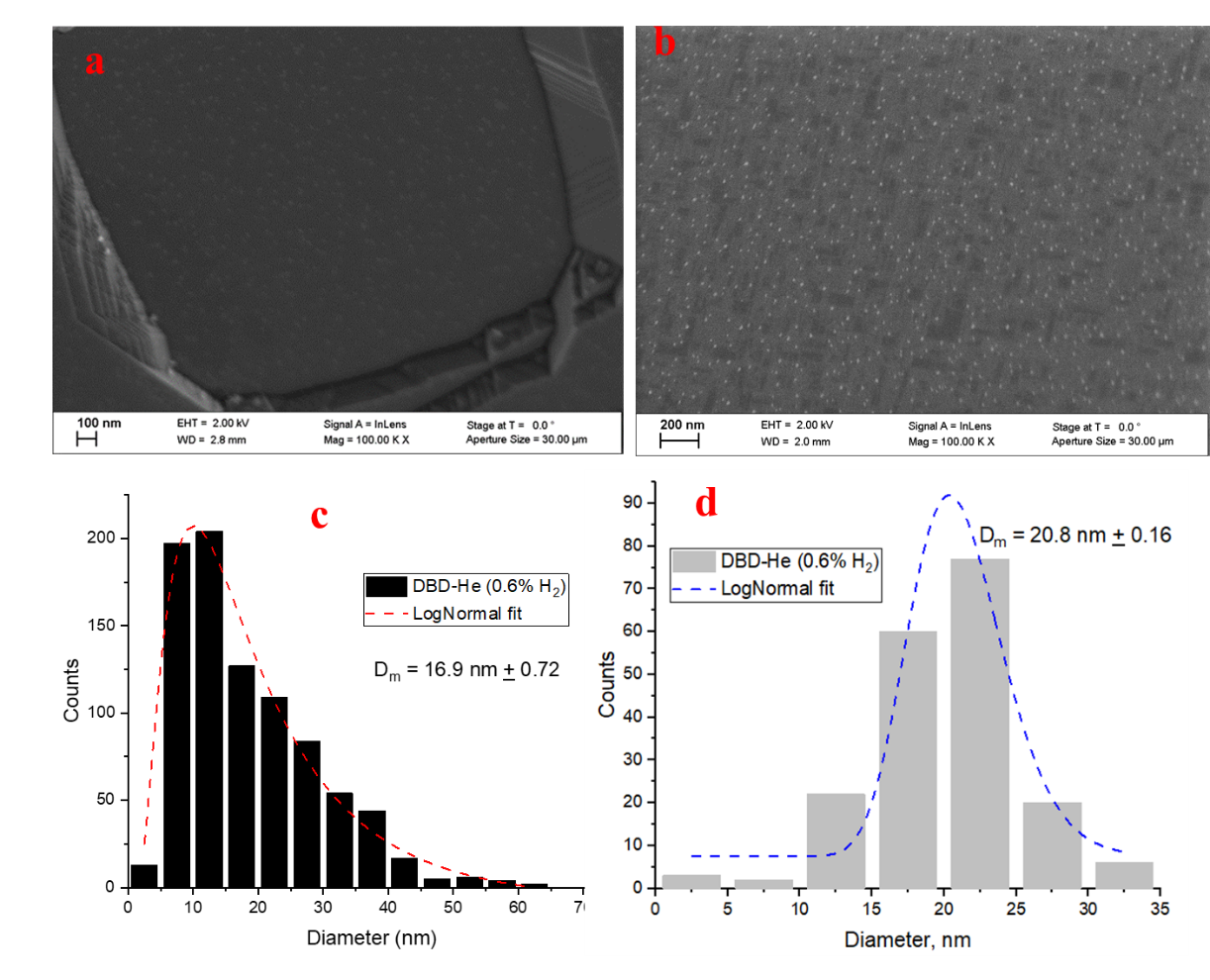


**Figure S4: (a-b)** FE-SEM images of **LCTN broken samples** treated in **He-DBD with 0.6 % hydrogen**; **(c-d)** the corresponding size distribution of exsolved NPs.

**
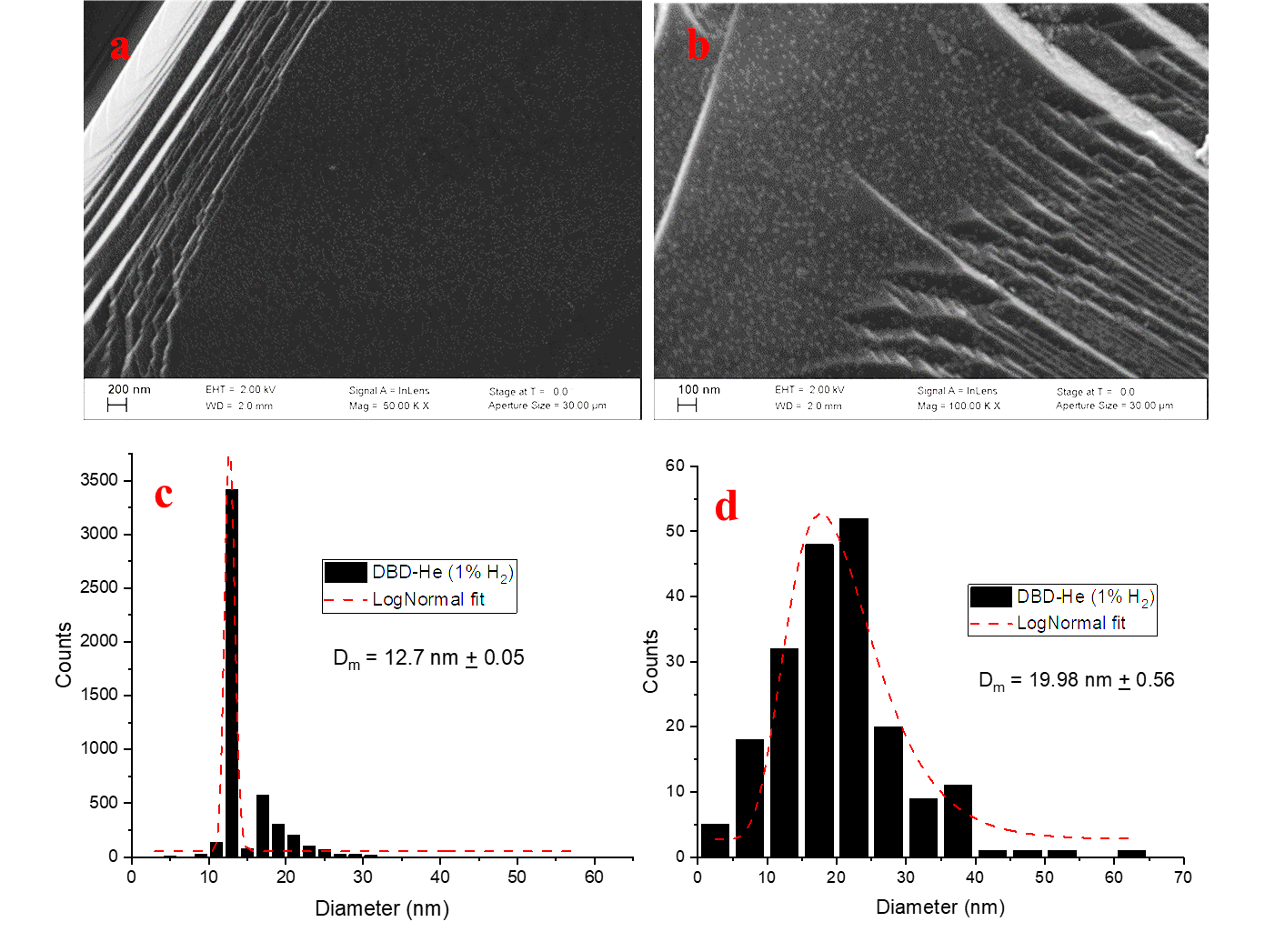
**

**Figure S5: (a-b)** FE-SEM images of **LCTN broken samples** treated in **He-DBD with 1 % hydrogen**; **(c-d)** the corresponding size distribution of exsolved NPs.

**
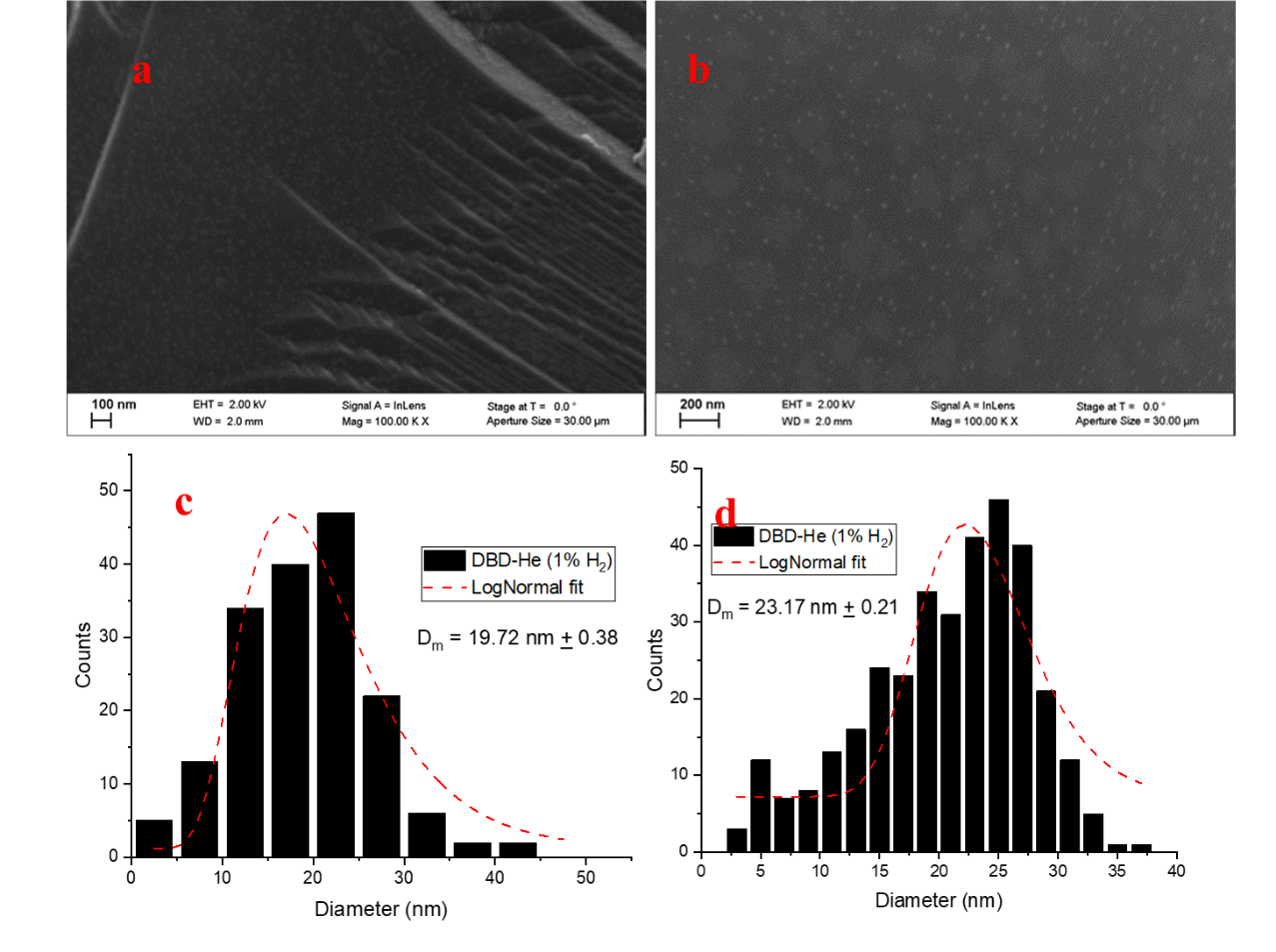
**

**Figure S6: (a-b)** FE-SEM images of **LCTN broken samples** treated in **He-DBD with 1 % hydrogen**; **(c-d)** the corresponding size distribution of exsolved NPs.

**
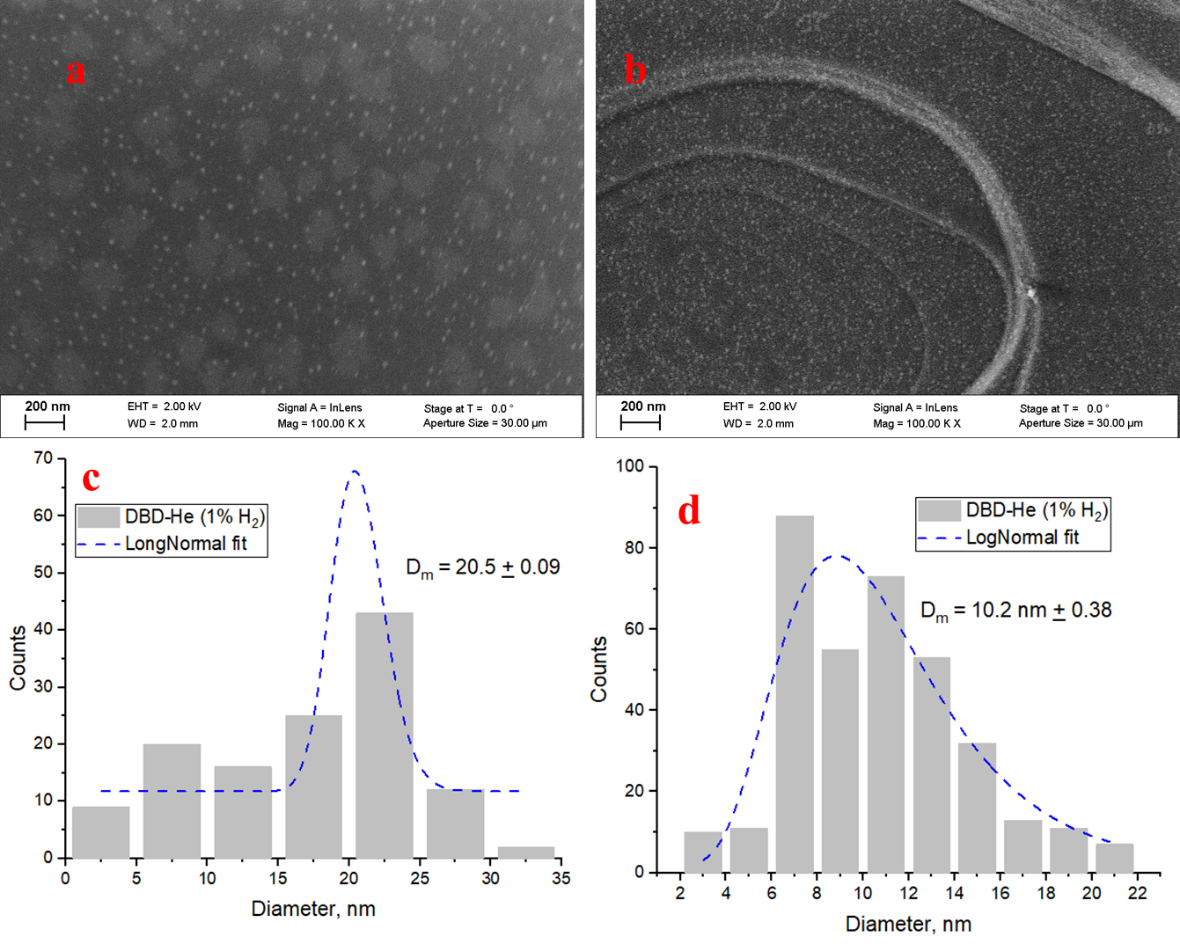
**

**Figure S7: (a-b)** FE-SEM images of **LCTN broken samples** treated in **He-DBD with 1 % hydrogen**; **(c-d)** the corresponding size distribution of exsolved NPs.


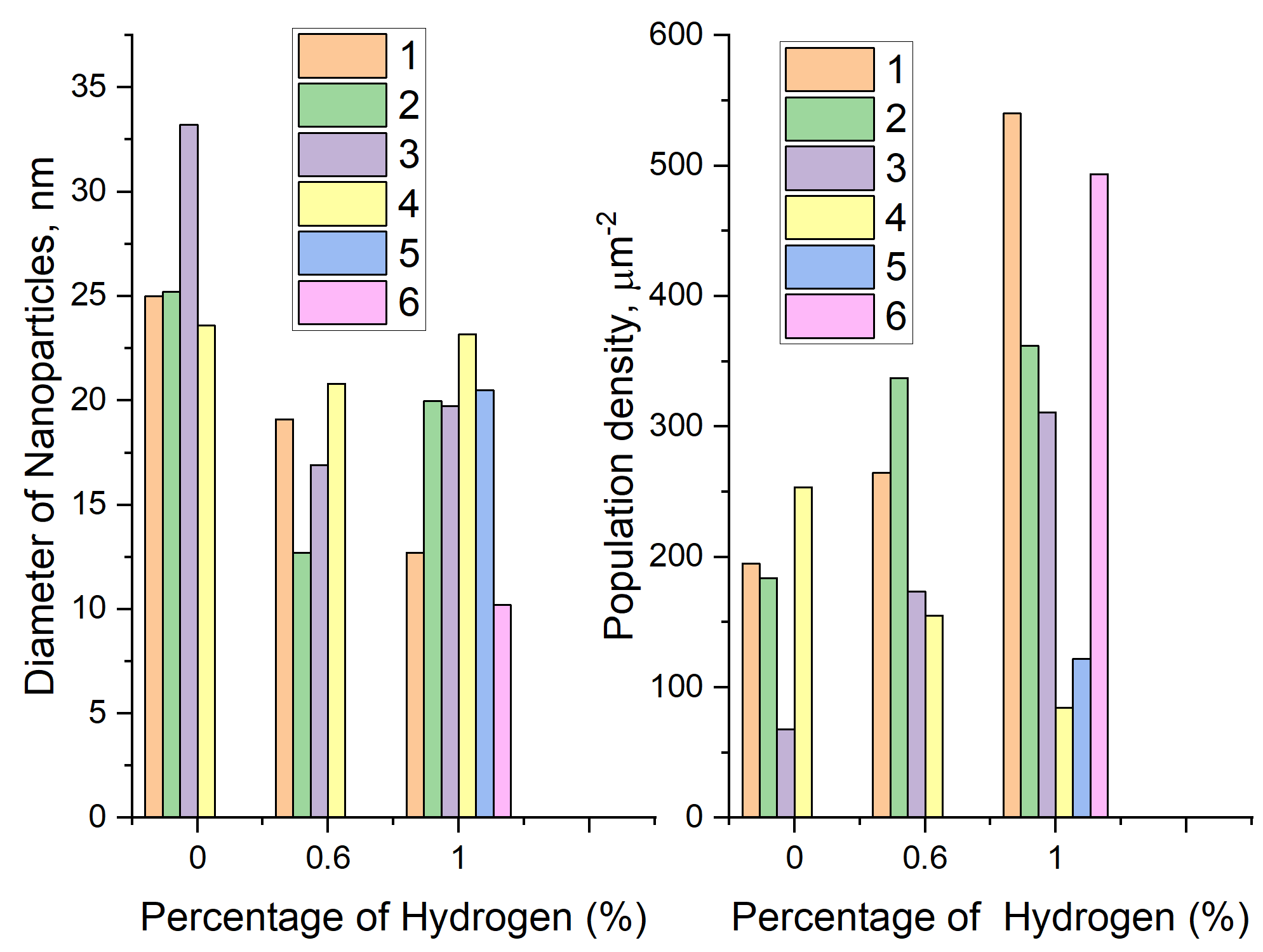


**a**

**b**

**Figure S8: (a-b)** A summary of size distribution and population densities of **LCTN broken samples** with increasing hydrogen concentration **from 0% to 1 % in the He-DBD**. The numbering 1-6 represents image number taken for image processing in ImageJ software.

**
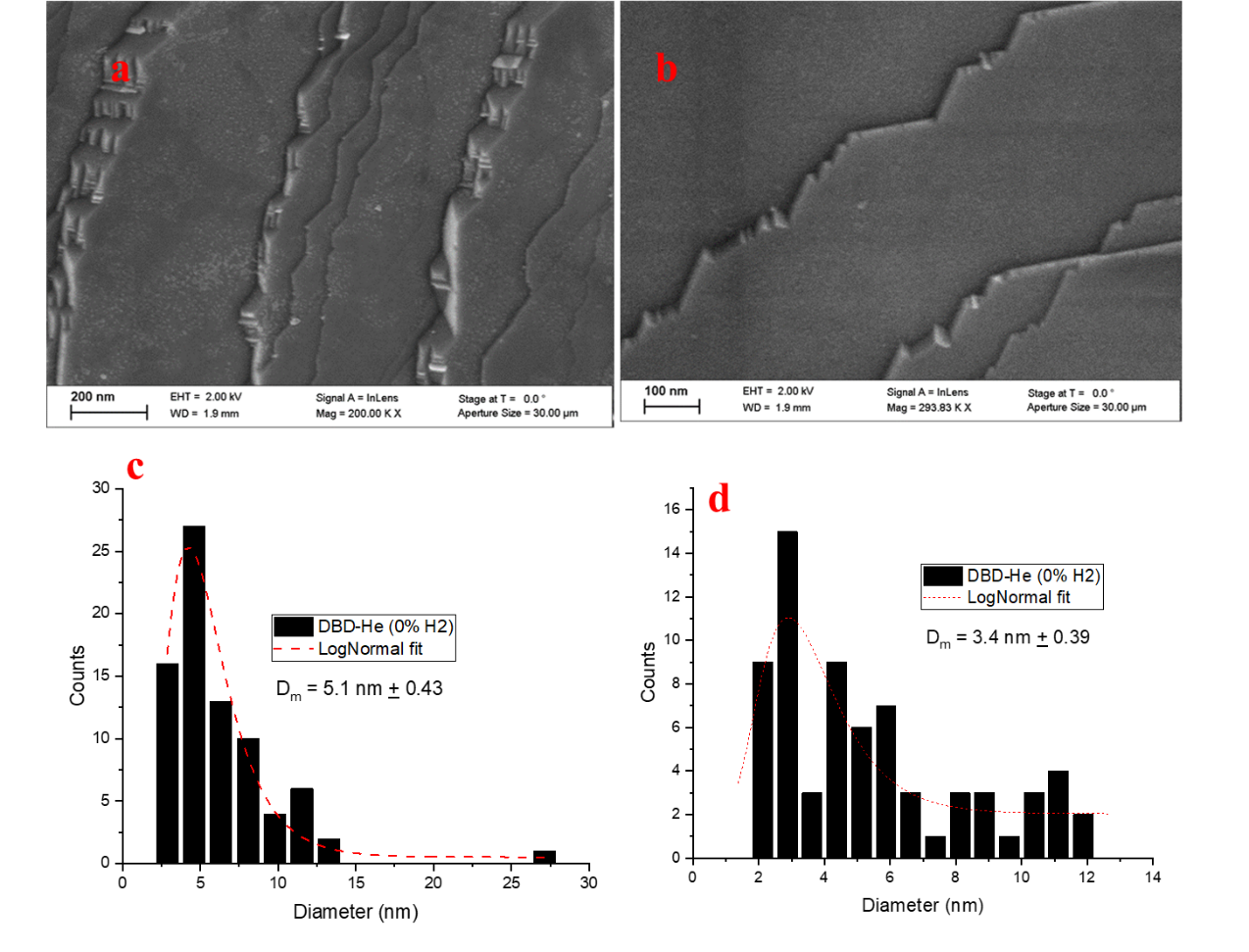
**

**Figure S9: (a-b)** FE-SEM images of **LCTN full pellets** samples treated in **He-DBD with 0 % hydrogen**; **(c-d)** the corresponding size distribution of exsolved NPs.

**
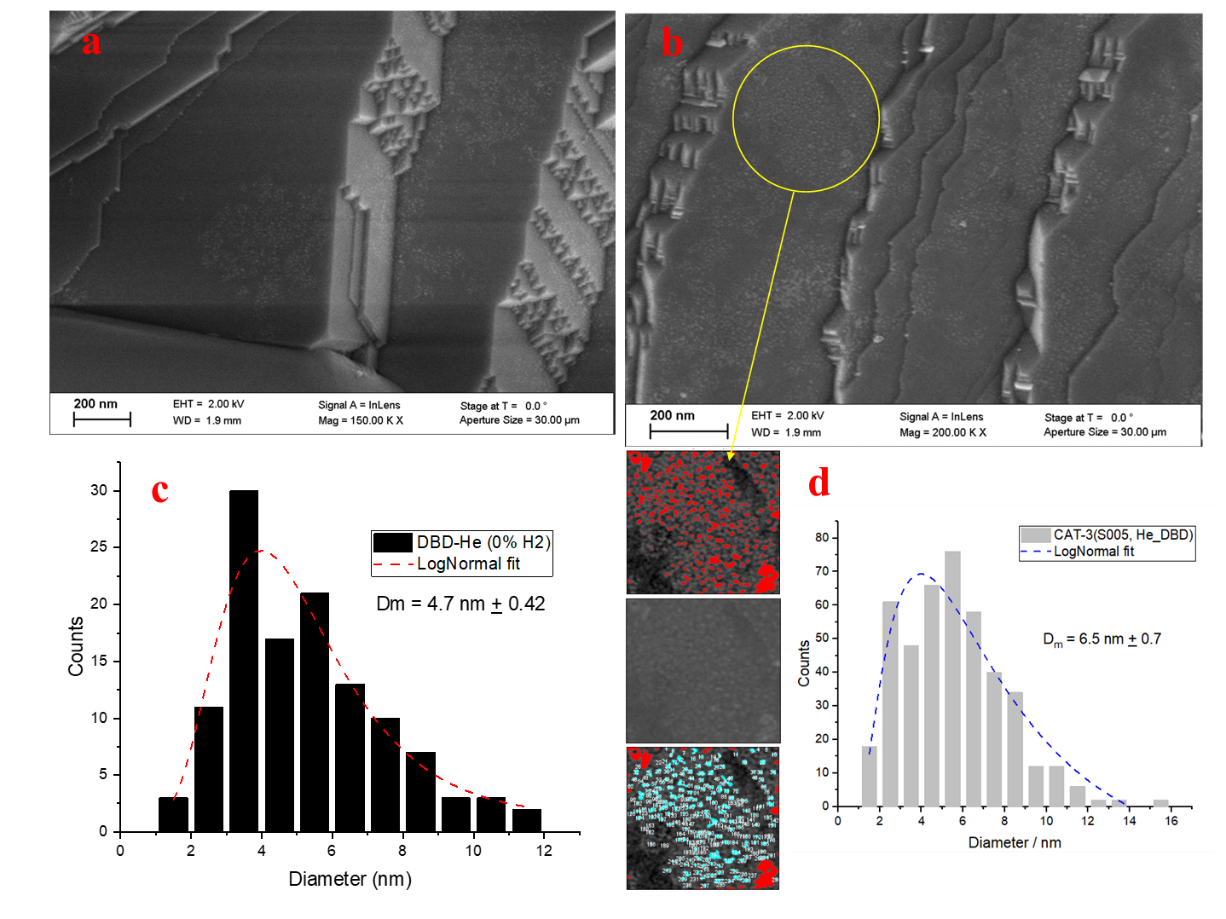
**

**Figure S10: (a-b)** FE-SEM images of **LCTN full pellets** samples treated in **He-DBD with 0 % hydrogen**; **(c-d)** the corresponding size distribution of exsolved NPs.

**
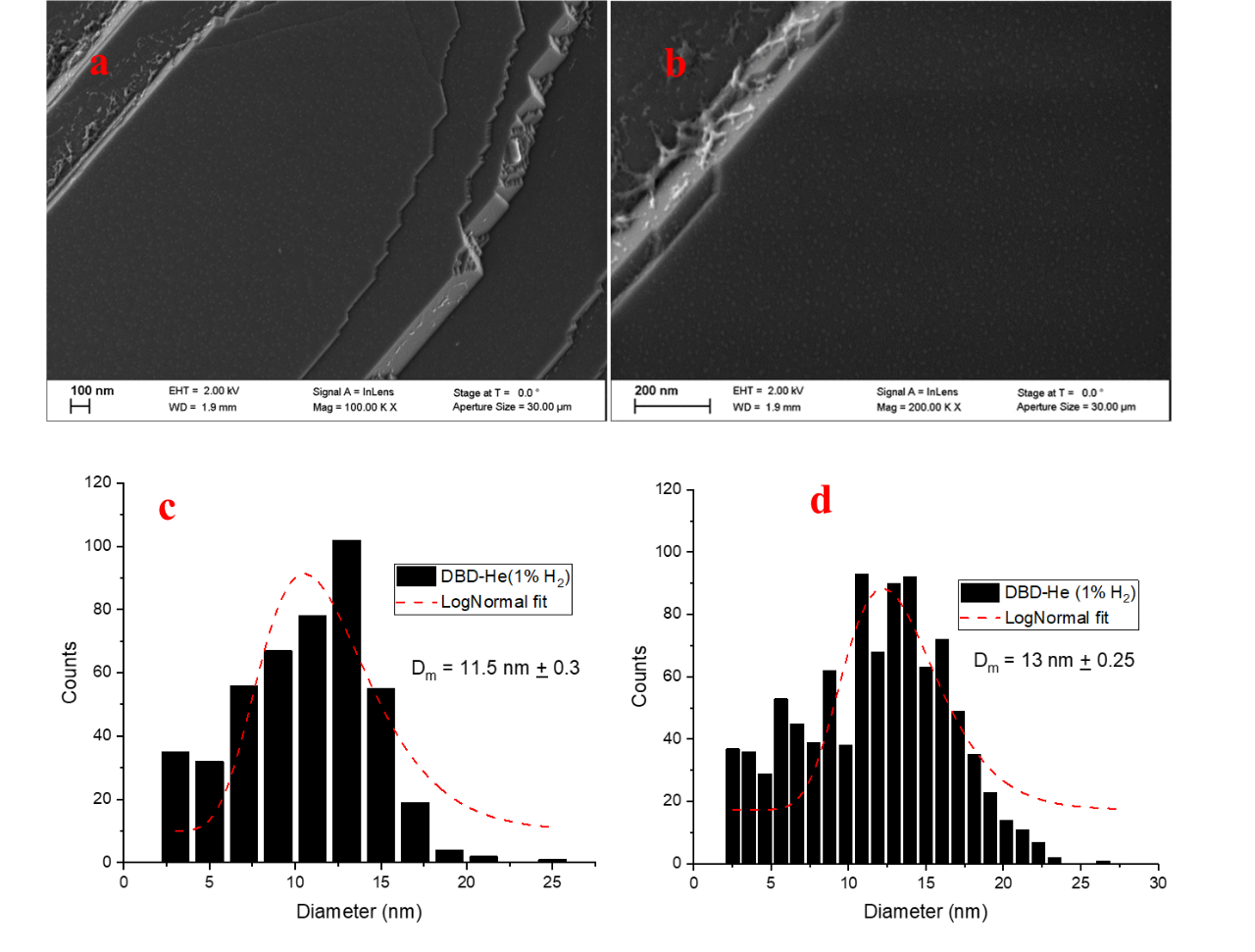
**

**Figure S11: (a-b)** FE-SEM images of **LCTN full pellets** samples treated in **He-DBD with 1 % hydrogen**; **(c-d)** the corresponding size distribution of exsolved NPs.


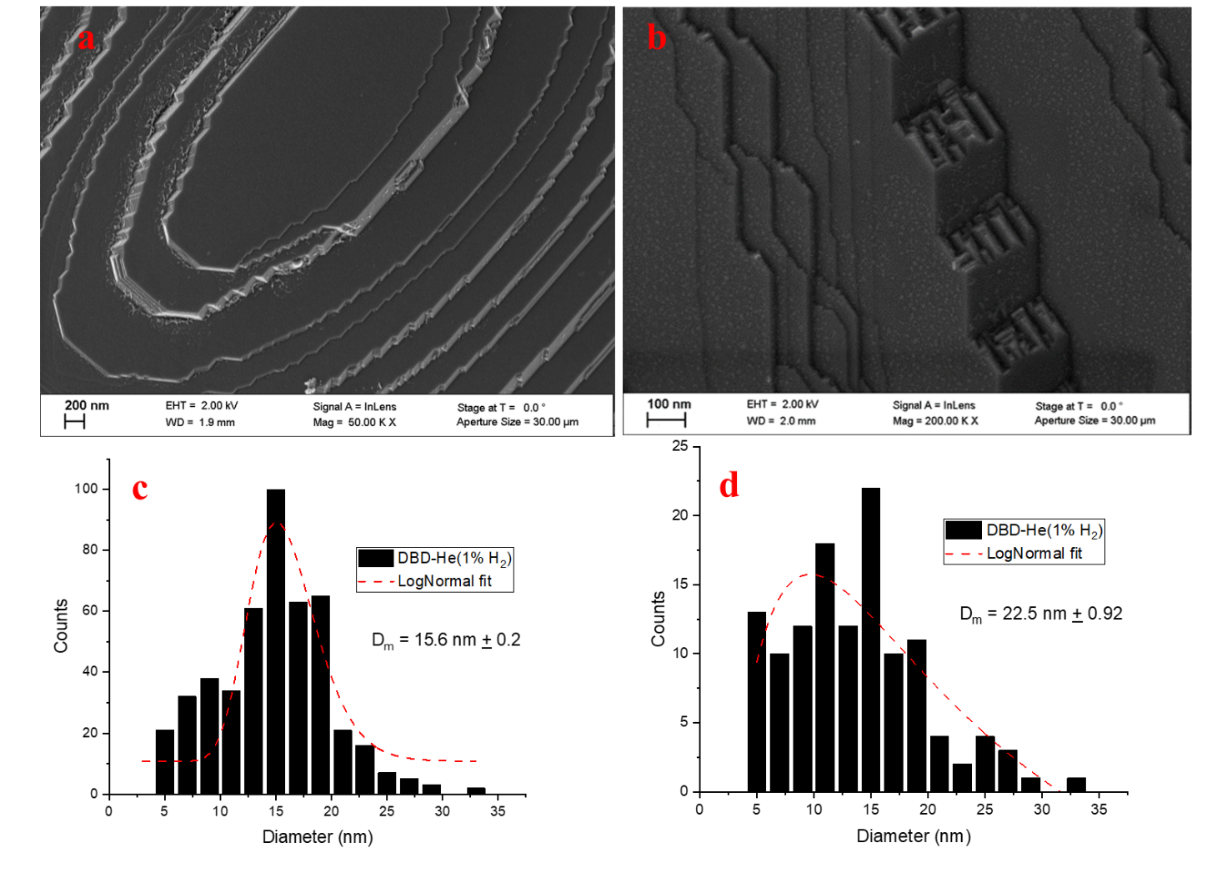


**Figure S12: (a-b)** FE-SEM images of **LCTN full pellets** samples treated in **He-DBD with 1 % hydrogen**; **(c-d)** the corresponding size distribution of exsolved NPs.


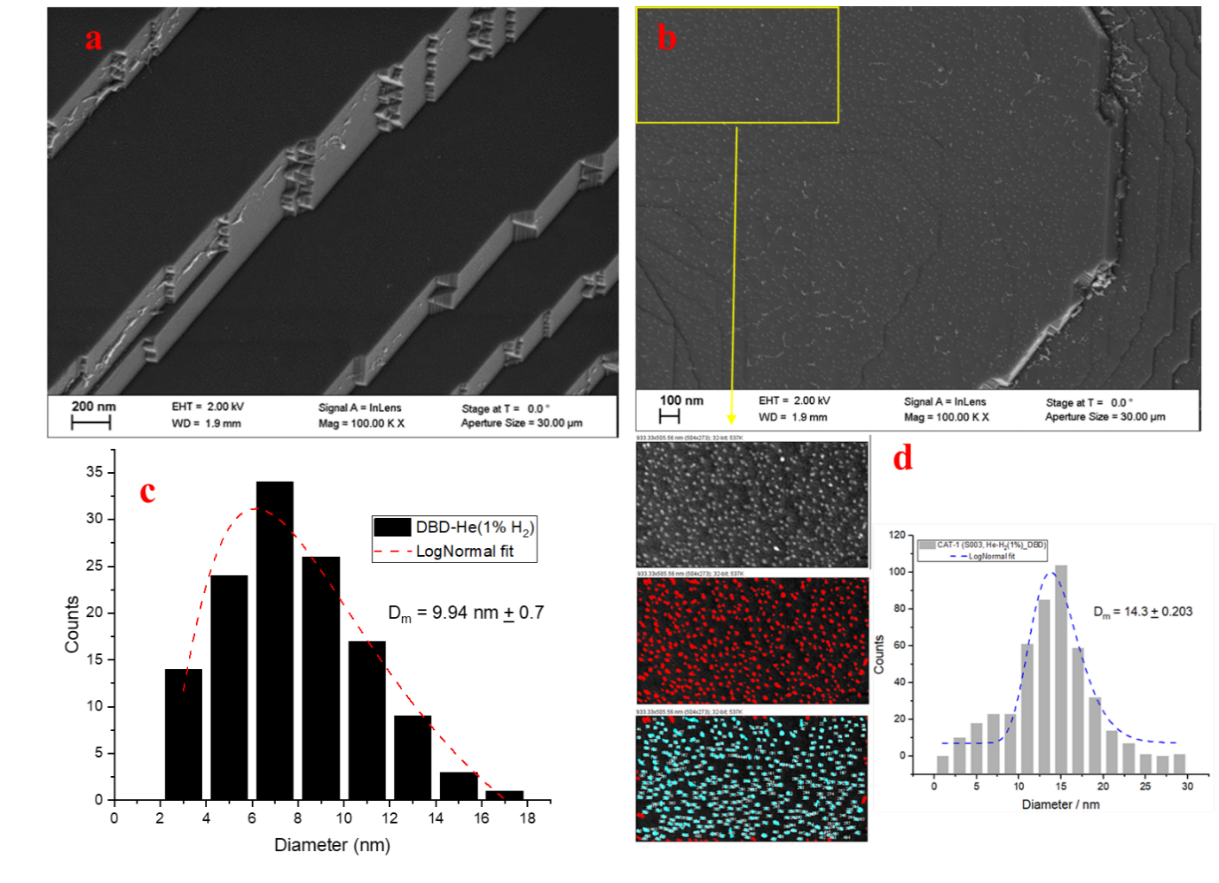


**Figure S13: (a-b)** FE-SEM images of **LCTN full pellets** samples treated in **He-DBD with 1 % hydrogen**; **(c-d)** the corresponding size distribution of exsolved NPs.


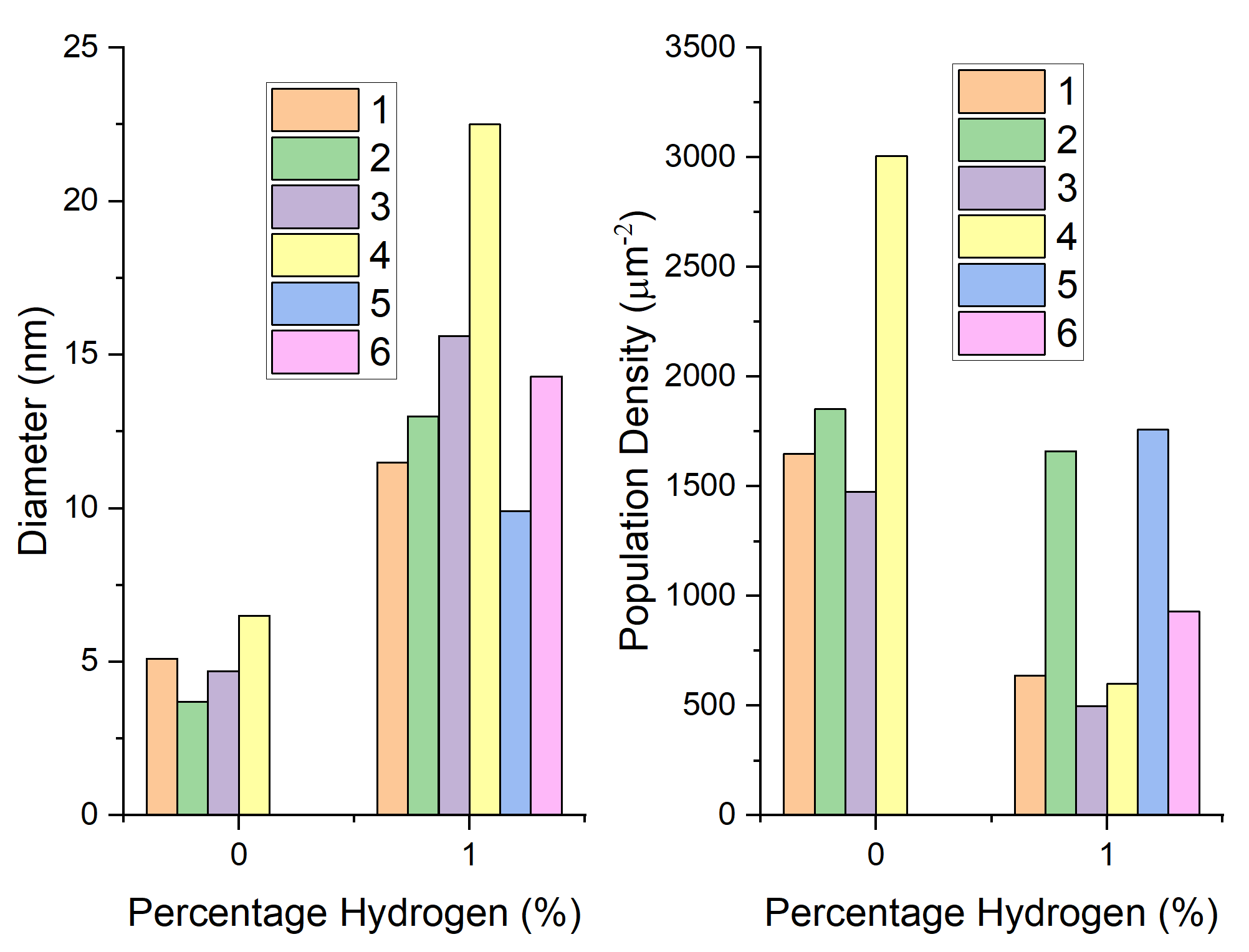


**b**

**a**

**Figure S14: (a-b)** A summary of size distribution and population densities of exsolved NPs in **LCTN-full pellets** with increasing hydrogen concentration **from 0% to 1 % in the He-DBD**. The numbering 1-6 represents image number taken for image processing in ImageJ software.

**SI-3: Additional materials characterization relevant to broken pellets**

**Table S1:** Atomic concentration of elements presents in perovskites oxides sample before and after the plasma treatment. These were determined from the high-resolution spectra after subtracting a Shirley-type background, using the Scofield sensitivity factors set in the MultiPak software. Impurities related to C, Si, Na, K, Ba and F are also sometime detected, which can be occasionally observed in some samples through XPS and XRD. To note that the atomic concentrations show a degree of sample-to-sample variability in the surface composition.

| **Sample** | **La (%)** | **Ca (%)** | **Ti (%)** | **Ni (%)** | **O (%)** |
| --- | --- | --- | --- | --- | --- |
| pristine | 5.3 | 5.1 | 11.4 | 1.1 | 77.1 |
| 0.0% H_2_ | 5.9 | 10.3 | 14.3 | 0.47 | 69.0 |
| 0.6% H_2_ | 5.3 | 9.3 | 13.7 | 0.59 | 71.1 |
| 1.0% H_2_ | 7.0 | 10.2 | 12.8 | 0.60 | 69.3 |

*
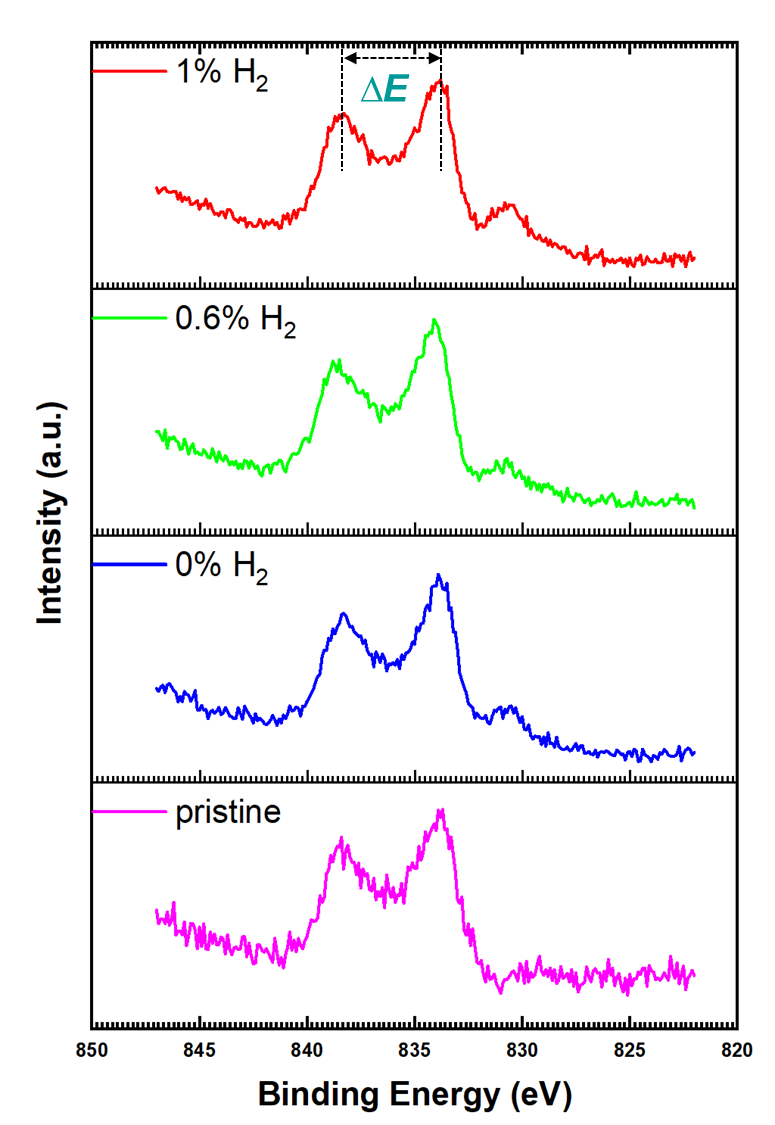
*

**Figure S15:** High-resolution XPS La 3d_5/2_ signals for pristine and DBD treated samples with 0% H_2_, 0.6% H_2_ and 1% H_2_ in the gas feed mixture, respectively. The splitting of the La 3d_5/2_ doublet is also highlighted (**∆**E = 4.6 eV) for all samples. This value is in agreement with the literature on lanthanum oxide (La_2_O_3_). It is worth mentioning that the splitting for lanthanum oxide (La_2_O_3_), lanthanum hydroxides and lanthanum carbonates have been reported to be around 4.6 eV, 3.9 eV and 3.5 eV, respectively.^2^ In our samples, the difference in the splitting is not significant and are very similar or closer to the value for lanthanum oxide (La_2_O_3_).


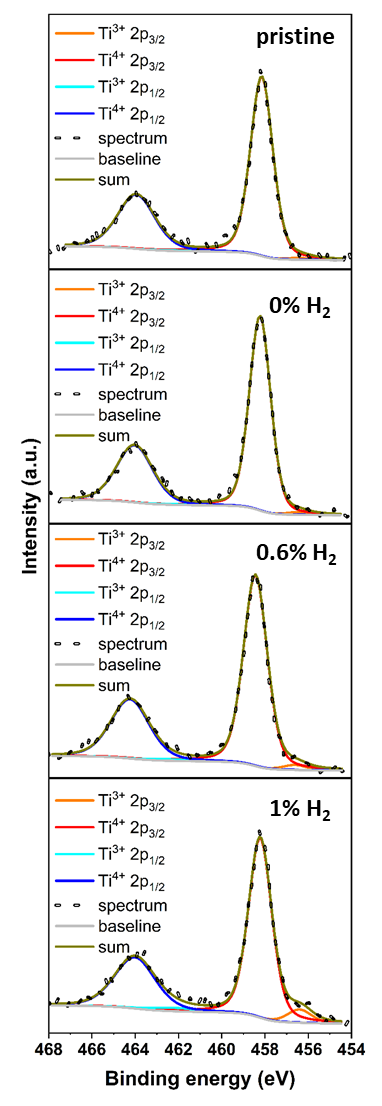


**Figure S16:** High-resolution XPS of Ti *2p* deconvoluted into Ti^4+^ and Ti^3+^. The Ti^3+^ peak increased with increasing the concentration of hydrogen in the feed mixture of the DBD.


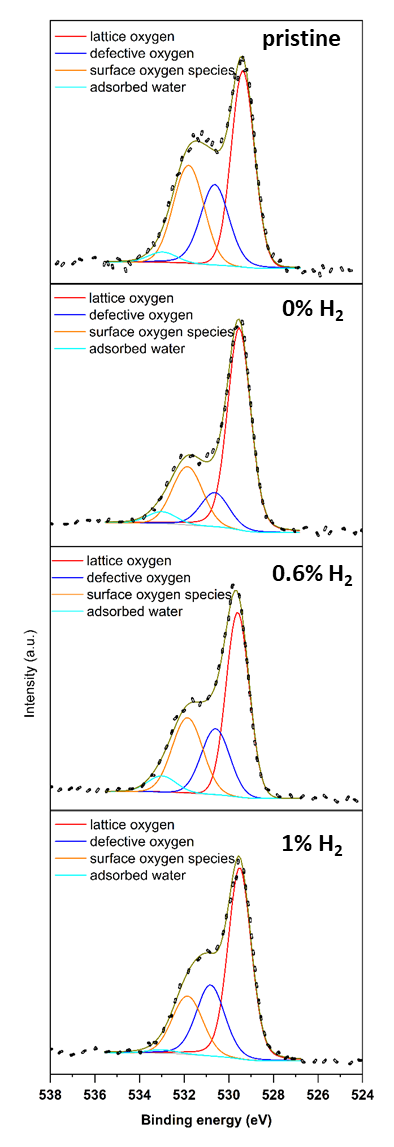


**Figure S17:** **(a-d)** High-resolution XPS of O *1s* deconvoluted into lattice oxygen, defective oxygen (oxygen vacancies), surface oxygen species and adsorbed water present in the pristine and He-DBD treated samples with 0% H_2_, 0.6% H_2_ and 1% H_2_ respectively.

**Table S2:** Binding energy positions of the curve-fitting components of the XPS Ni 3p_3/2_, Ti 3s and Ti 2p_3/2_ signals that were used to obtain the results reported in Table 1. No significant peak shifts were observed after plasma exsolution.

| Sample | Ni^2+^ 3p_3/2_  (eV) | Ni^0^ 3p_3/2_  (eV) | Ti^4+^ 3s  (eV) | Ti^3+^ 3s  (eV) | Ti^4+^ 2p_3/2_  (eV) | Ti^3+^ 2p_3/2_  (eV) |
| --- | --- | --- | --- | --- | --- | --- |
| Pristine | 67.7 | - | 62.0 | 59.4 | 458.1 | 456.4 |
| 0.0% H_2_ | 67.5 | 65.4 | 62.2 | 59.5 | 458.2 | 456.5 |
| 0.6% H_2_ | 67.8 | 65.5 | 62.2 | 59.6 | 458.4 | 456.6 |
| 1.0% H_2_ | 67.7 | 65.6 | 62.2 | 59.6 | 458.2 | 456.4 |


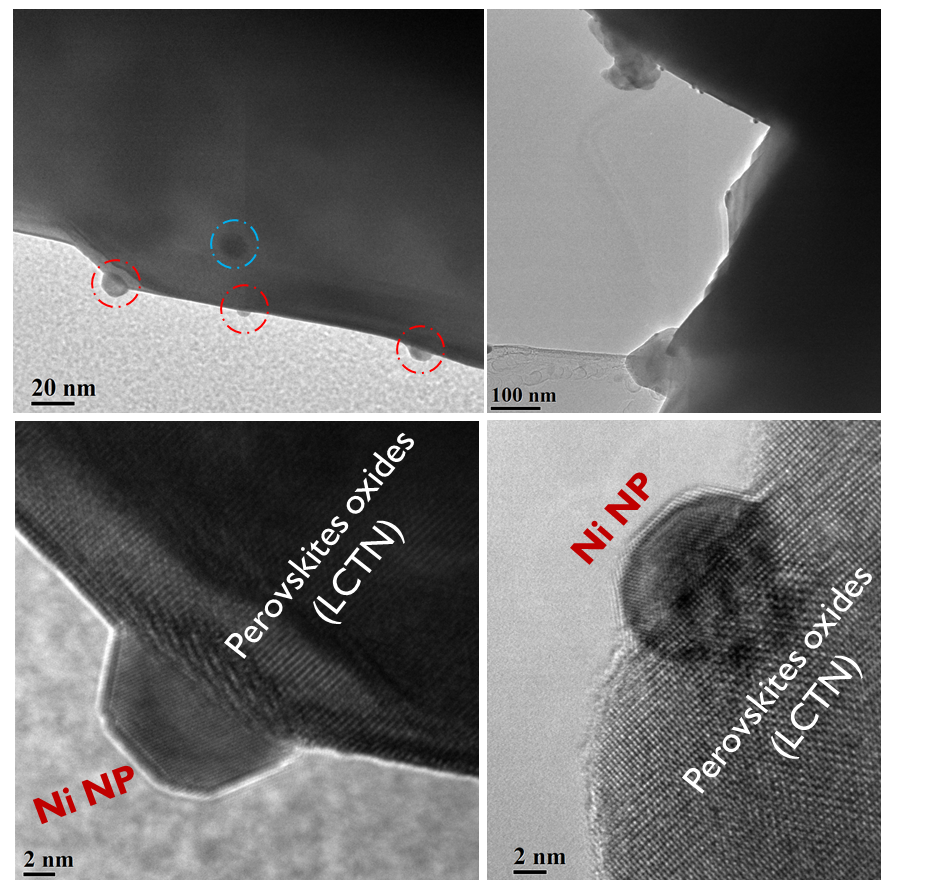


c

d

a

b

**Figure S18: (a-b)** Low resolution transmission electron microscopy (TEM) of exsolved Ni NPs on the LCTN surface treated in 1% H_2_ DBD; (c-d) HR-TEM images of the exsolved Ni NPs showing faceted, socketed nature.


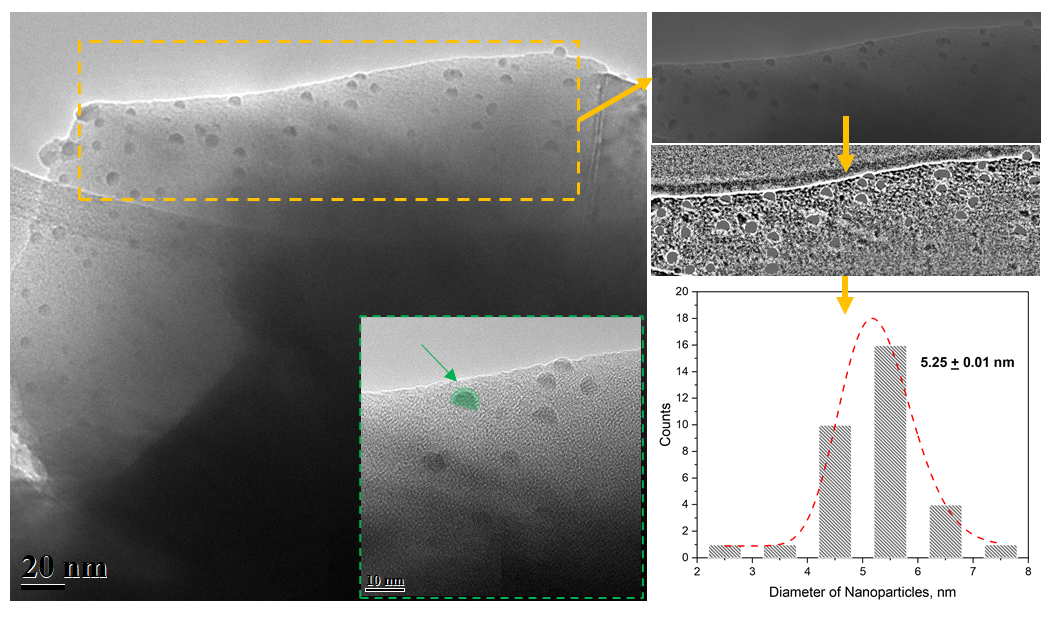


d

c

b

a

**Figure S19: (a)** Transmission electron microscopy (TEM) of exsolved Ni NPs on the LCTN surface, treated in 1% H_2_ DBD, showing hemispheric shape i.e., partially emerged, and socketed (inset); (b) Ni NPs from the selected rectangular area in (a); (c) selection of Ni NPs in Image J for creating a size distribution; (d) bar-chart diagram showing the size distribution of Ni NPs showing 5.25 nm. The dashed red curve represents the log-Normal fit.

**SI-4: Additional materials characterization relevant to full pellets**

The samples for catalytic testing required large and preferably circular pellets. Hence, full pellet samples were treated in DBD fed with pure helium and with He/ 1.0% H_2_ mixture. The size of the samples is one of the parameters that affect the exsolution process, the plasma treatment time was increased from 10 min (broken pellets) to 15 min. Figure S21a-b shows SEM images of LCNT samples treated in plasma without hydrogen and with hydrogen (1% H_2_) revealing bright spots relating to exsolved Ni NPs. It can be seen from Figure S20a that the NPs are exsolved just by He-plasma which resulted in very small NPs with extremely high population densities. The NPs average diameter is larger when hydrogen (1%) is used in the plasma as shown in Figure S21b. Figure S20c represents a parallel plot of the changes in diameter of Ni NPs and population densities at both 0% H_2_ and 1% H_2_. The average diameter of NPs after He-DBD was around 5 nm while it was ~14 nm when 1% H_2_ was used (Figures S7-12). The population densities on the other hand were ~2000 µm^-2^ for He-only and ~1000 µm^-2^ when 1% hydrogen was used (Figure S21c). Figure S20d shows the XRD of LCTN full pellet sample revealing the main perovskites crystal structure.


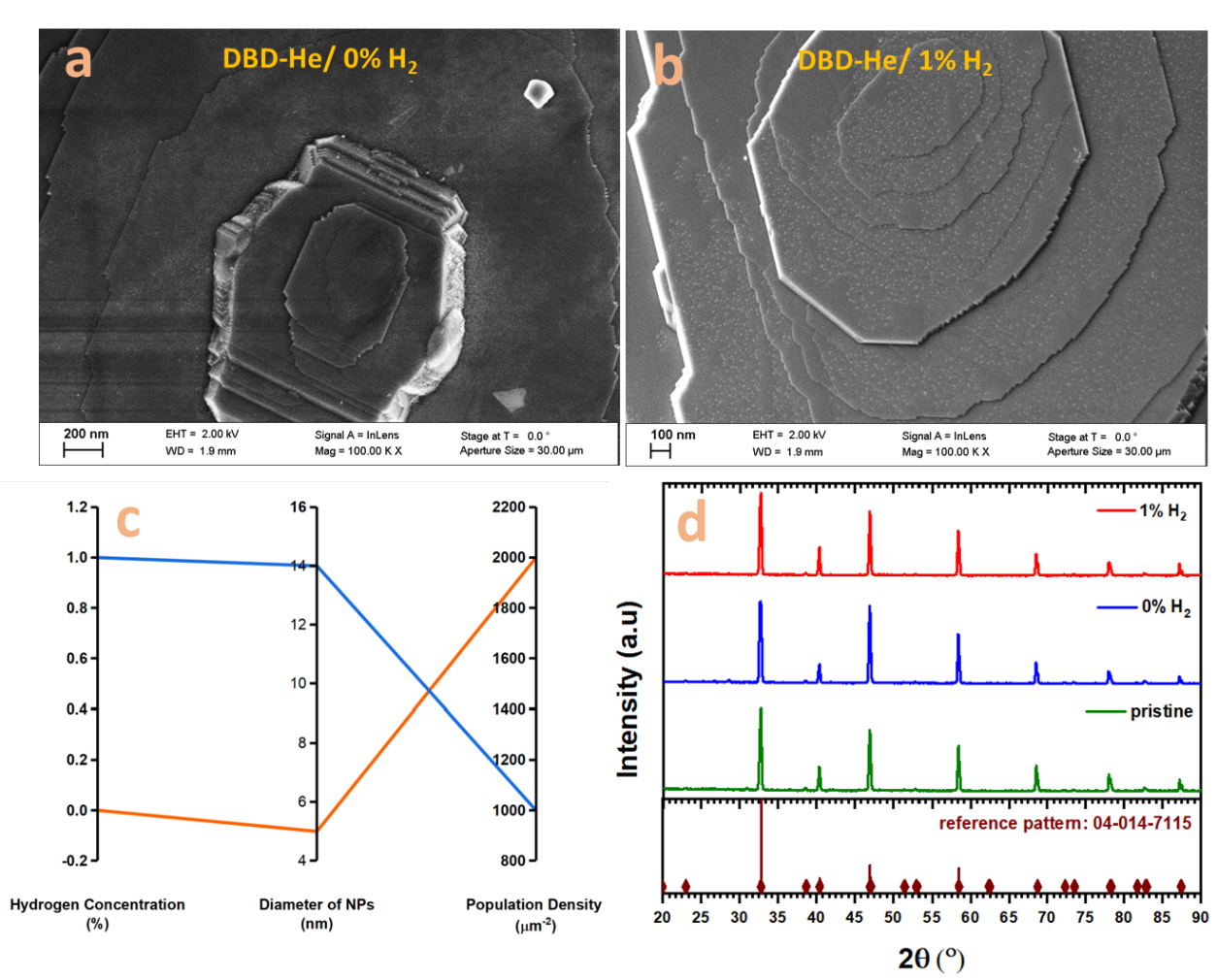


**Figure S20:** **(a-b)** Field-emission scanning electron micrographs (FE-SEM) of La_0.43_Ca_0.37_Ni_0.06_Ti_0.94_O_2.955_ (LCTN-full pellets) treated in DBD fed with He and He/1% H_2_, respectively; **(c)** A parallel plot of changes in the size of Ni NPs and population density, at 0% H_2_ and 1% H_2_ respectively. **(d)** X-ray Diffraction of LCTN samples confirming orthorhombic structure.


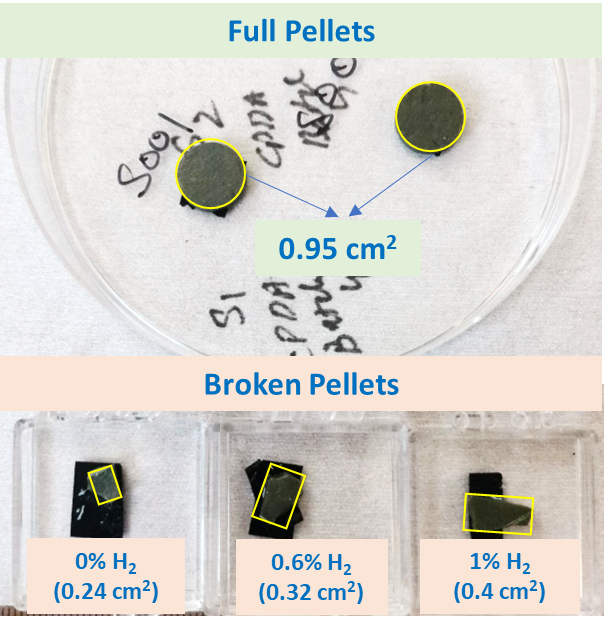


**Figure S21:** A photograph of samples taken just to show an estimated area exposed to the plasma in DBD. The area of full pellets was about 0.95 cm^2^ in all experimental conditions.

**SI-5: Calculations of exsolution depth and exsolved Ni atoms**

The volume of a Ni nanoparticle (NP) is given by

$$V_{Ni-NP}=\frac{4}{3}\pi\left( \frac{d}{2} \right)^{3}$$

where *d* is the NP diameter and the number of Ni atoms in a NP is

$$A_{Ni-NP}=\frac{\left( \rho_{Ni}V_{Ni-NP} \right)}{A_{Ni}\times\left( 1.66\times{10}^{-27} \right)}$$

with *ρ*_Ni_ = 8900 kg m^-3^ the mass density of Ni, *A*_Ni_ = 58.69 the atomic mass of Ni and 1.66 x 10^-27^ kg is 1 mass unit.

We now determine the number of Ni ions exsolved per surface area

$$A_{ex-Ni/SA}=A_{Ni-NP}\times{SD}_{Ni-NP}$$

where SD_Ni-NP_ is the experimentally determined density of nanoparticles on the surface. The number of Ni ions available for exsolution per nm depth and per surface area is given by

$${Ni}_{ions/SD}=\frac{0.06}{0.547\times0.547\times0.772}=0.259 \mathrm{nm}^{-3}$$

where 0.06 is the Ni atom concentration and 0.547 nm x 0.547 nm x 0.772 nm are the unit cell parameters determined by Rietveld refinement of a pristine LCTN sample. The calculation for the depth (*D*) of exsolution is given by the following equation:

$$D=\frac{A_{ex-Ni/SA}}{{Ni}_{ions/SD}}$$

The results of these calculations are summarized in Table 3.

**Table S3**. Experimental and calculated parameters to determine the exsolution depth.

| Sample | Experimental | | Calculated | | | |
| --- | --- | --- | --- | --- | --- | --- |
|  | ***d***  **nm** | ***SD_Ni-NP_***  **µm^-2^** | ***V_Ni-NP_***  **nm^3^** | ***A_Ni-NP_*** | ***A_ex-Ni/_*_SA_**  **µm^-2^** | ***D***  **nm** |
| 0.0% H_2_ | 27 | 175 | 10306 | 9.41x10^5^ | 1.65x10^8^ | 634 |
| 0.6% H_2_ | 17 | 233 | 2758 | 2.52x10^5^ | 5.87x10^7^ | 226 |
| 1.0% H_2_ | 18 | 319 | 2903 | 2.65x10^5^ | 8.46x10^7^ | 326 |

Assuming oxidation of Ni to NiO NPs or NiO exsolution, the depth of exsolution can be calculated in a similar way as described above, by just including the values related to NiO such as density (6.67 g cm^-3^) and molecular mass (74.7 g mol^-1^). The depth of exsolution as a function of hydrogen concentration for Ni and NiO NPs has been plotted in Figure S22.


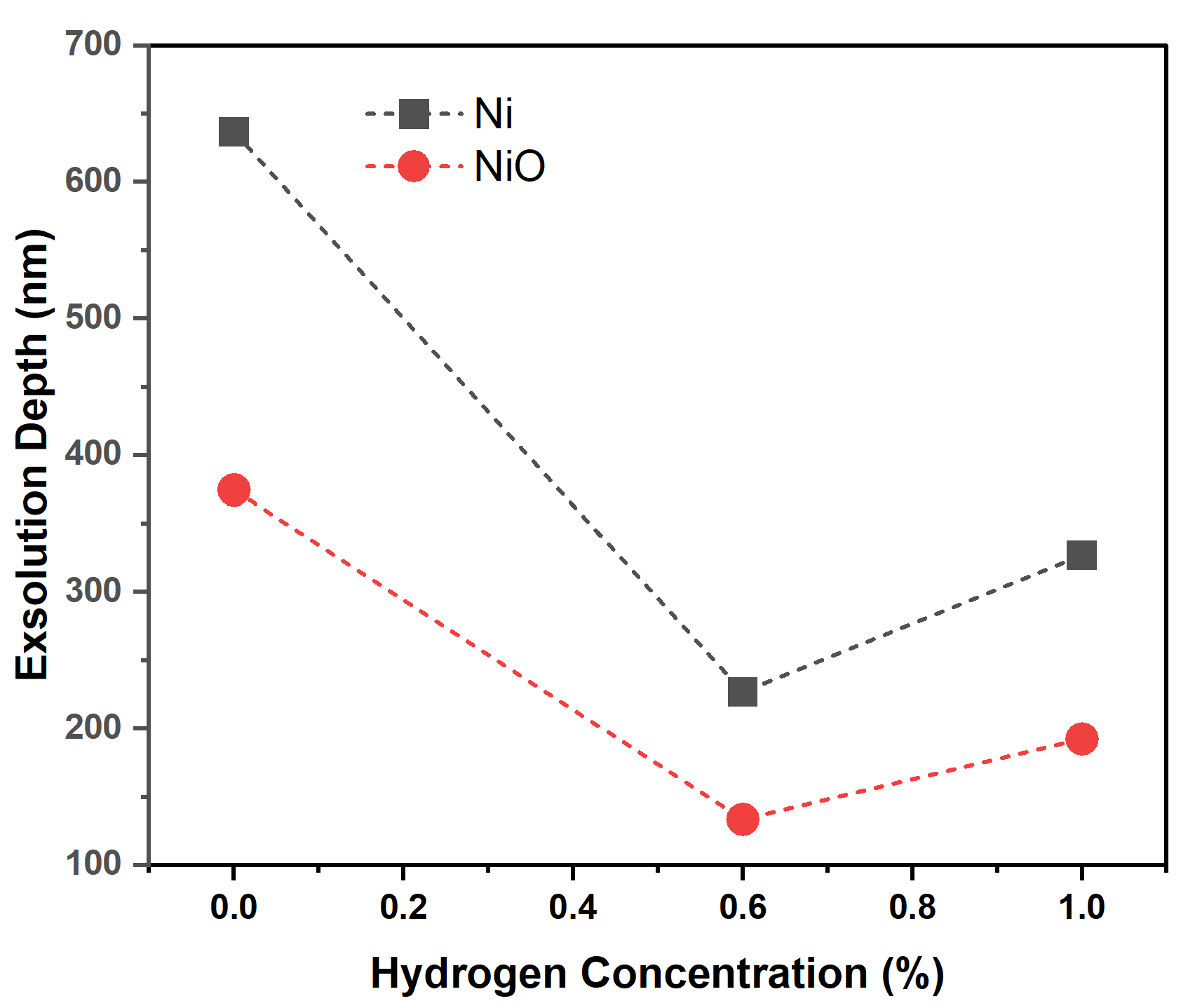


**Figure S22:** Exsolution depths (nm) estimated from the calculations assuming exsolution of pure Ni and NiO nanoparticles at different concentrations of hydrogen in the He-plasma.

**SI-6: Additional results in support of magnetic measurements**

Figure S23 shows example magnetisation data for a LCTN sample (not exfoliated) which has been plasma treated using He-DBD with 1% H_2_. In figure S23a the magnetic moment is plotted for a sample measured at 300 K, for applied fields with flux density over the range of $\pm5000$G (0.5 T). The dominant (linear) contribution to the signal is due to the Ni^2+^ paramagnetic signal from the host material, though there is evidence of hysteresis due to the exsolved metallic Ni around the origin. This latter contribution is however indiscernible in the data taken at 2 K shown in figure S23b, since it is completely swamped by the much larger paramagnetic signal at low temperature.


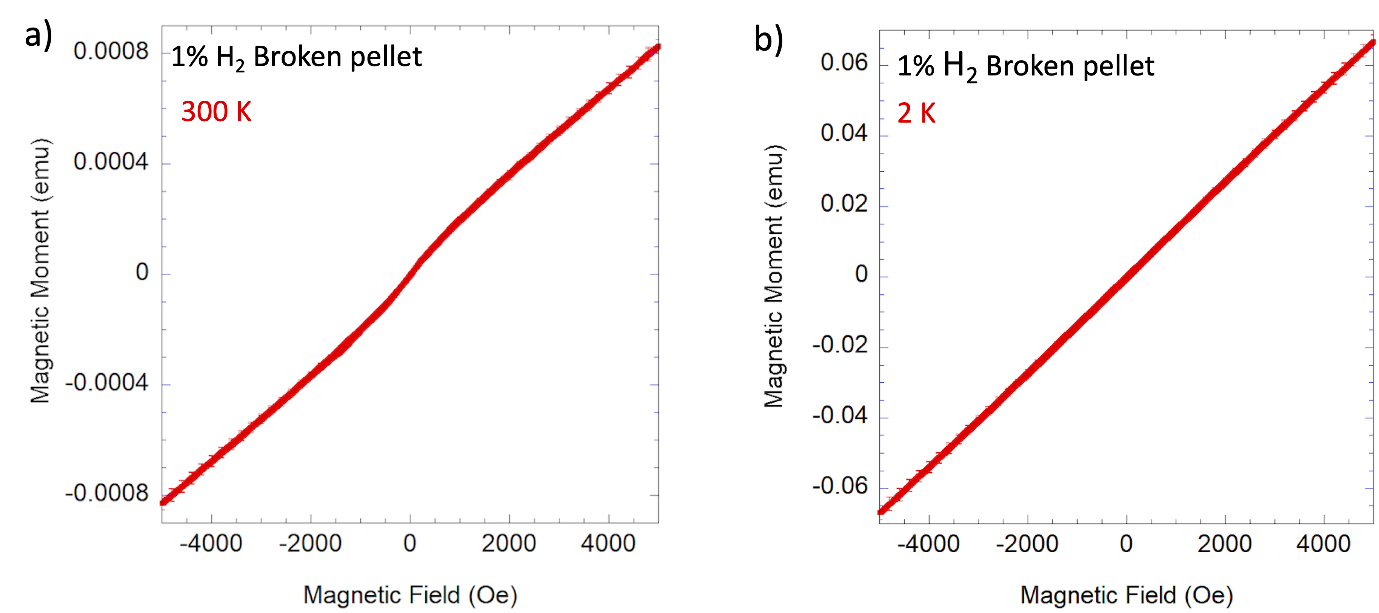


**Figure S23:** Magnetic moment as a function of applied field for samples after plasma treatment in He-DBD with 1% H_2_ measured at **(a)** 300 K and **(b)** 2 K.

In addition to a number of exfoliated LCTN samples taken from the upper surface of the pellets, exfoliated samples were also taken from the underside of the pellets which was not exposed to the plasma and not exsolved. Figure S24a shows a typical signal taken from the underside, after correcting for the dominant diamagnetic background, compared to a comparable signal taken from the upper surface. It can be seen that there is little signature of nanoparticle magnetism on the lower surface confirming the validity of the measurement from the upper surface. Finally it can be seen from figure S24b that there is some evidence of magnetic nanoparticles on the lower side of the broken pellet for the He-DBD 0% H_2_ sample, though significantly less than for the exposed upper surface. Further work is required to investigate systematically the degree of lower surface exsolution in all samples in relation to the position on the surface, which exfoliation techniques should make possible.


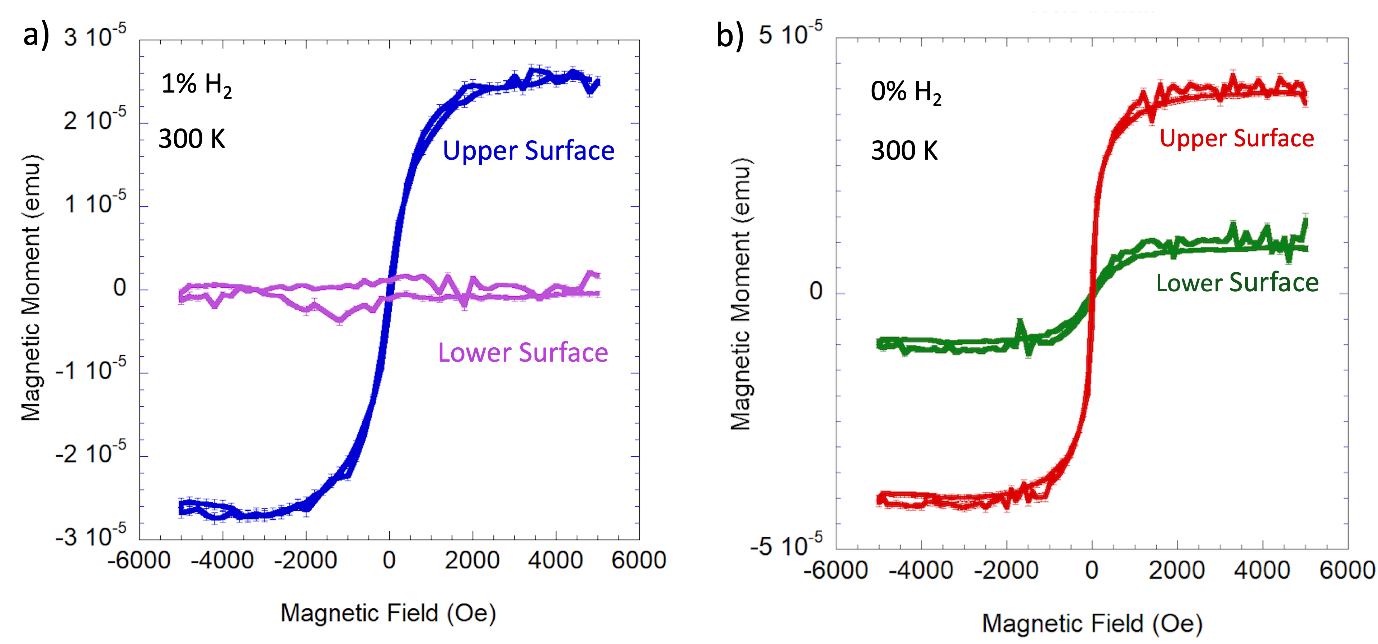


**Figure S24:** **(a)** Magnetic moment as a function of applied field for an exfoliated sample after plasma treatment in He-DBD with 1% H_2_ measured at 300 K. Comparison of exfoliation from the upper and lower surface of the sample. **(b)** Magnetic moment as a function of applied field for an exfoliated sample after plasma treatment in He-DBD with 0% H_2_ measured at 300 K. Comparison of exfoliation from the upper and lower surface of the sample.

**SI-7: Additional results in support of catalytic testing**

To demonstrate that the exsolved catalyst samples are capable of maintaining their performance during long-term operation, we have performed long-term stability tests under isothermal conditions for both the exsolved LCNT-He and impregnated LCT samples. The long-term stability tests were performed for both samples at 800 °C for the CH_4_ oxidation reaction. This reaction was selected as it is known that cocking is a challenge for Ni-based catalysts whereas the temperature of 800 °C will accelerate possible agglomeration of the Ni nanoparticles. The results of the long-term stability test for both catalysts can be seen in Figure S25.

**Figure S25:** Catalytic activity (in terms of CO and CO_2_ production rates from the CH_4_ oxidation reaction) as a function of time for the LCNT-He and LCT.

Figure S25 demonstrates the performance of the catalysts in terms of CO and CO_2_ production rates during the methane oxidation reaction for the LCNT-He and LCT catalysts versus time. It is clear that the production rate of the impregnated LCT catalyst decreases fast and the catalyst is severely deactivated after 1 hour of operation. In contrast the LCNT-He catalyst shows no measurable signs of deactivation for the whole duration of the (120 h) long-term stability test.

**SI-8: Additional XRD results and Rietveld Refinement**

Figure S26a shows the magnified plot of the XRD data shown in Figure 3 of the main manuscript. Slight variation in the peak position before and after the DBD plasma treatment can be seen in the Figure S26a. The first three intense peak were chosen for calculating the FWHM. The changes in the FWHM for different LCTN samples are shown in Figure S26b.

Rietveld refinement of the XRD data were performed using the open GSAS-II software and fitting to orthorhombic structure (space group: Pbnm).^3^ Figure S27a-d shows the Rietveld refinement of the XRD of the LCTN samples before and after plasma treatment (0%H_2_, 0.6%H_2_ and 1%H_2_). The details about the cell parameters and cell volume are given in Table S4. There are small changes in the values of the cell parameters and cell volume compared to as untreated LCTN sample. The cell volume of untreated LCTN is decreased with increasing the hydrogen concentration in the DBD plasma.

**
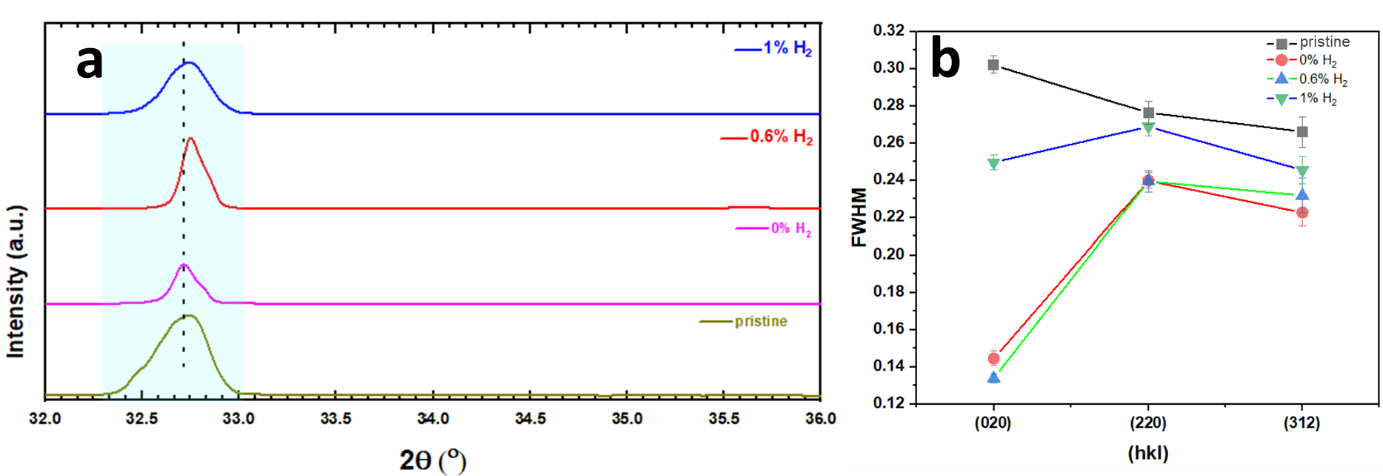
**

**Figure S26:** (a) A magnified XRD data of the most intense peak of LCTN showing slight variations in their peak position before and after plasma treatment; (b) Variations in the FWHM calculated from the first three strongest peaks in the XRD of LCTN.


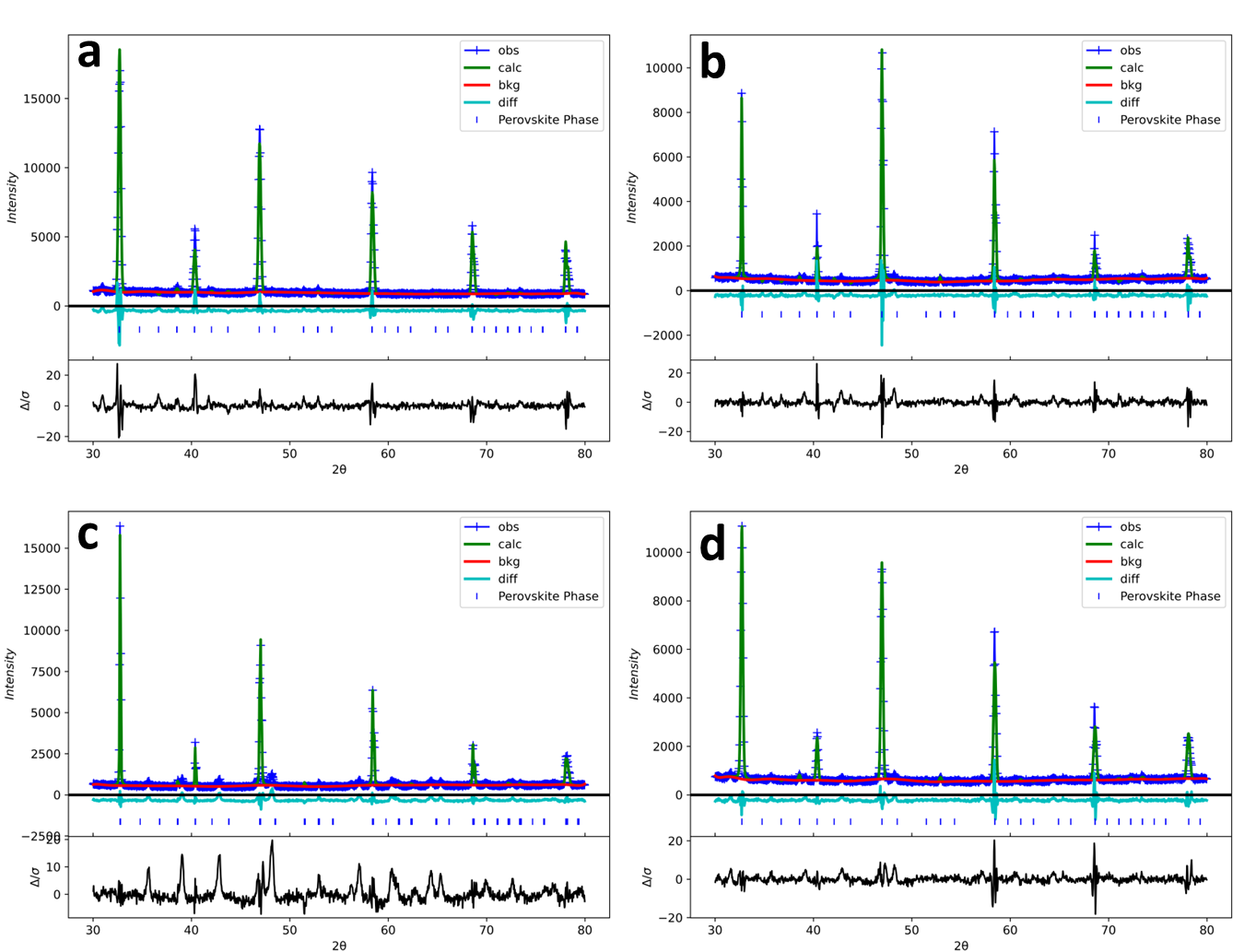


**Figure S27:** (a-d) Rietveld refinement of the XRD patterns of pristine, 0% H_2_ 0.6% H_2_ and 1% H_2_ LCTN samples respectively.

**Table S4:** Cell parameters obtained from the Rietveld refinement of LCTN samples.


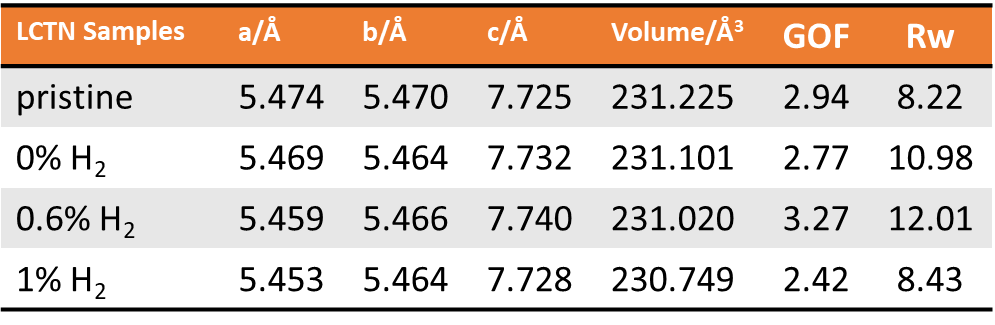


The Rietveld refinement of the XRD data of LCTN-full pellets (Figure S20) are shown in Figure S28 and the summary of the cell parameters and cell volumes is shown in Table S5. The cell volume in pristine LCTN sample reduced from 231.594 Å^3^ to 231.262 Å^3^ when treated under 0% H_2_ DBD plasma. Interestingly, the cell volume is increased to 231.448 Å^3^ after treatment under 1%H_2_ DBD plasma. Such variations in the cell parameters and cell dimensions could be because of the creation of oxygen vacancies.


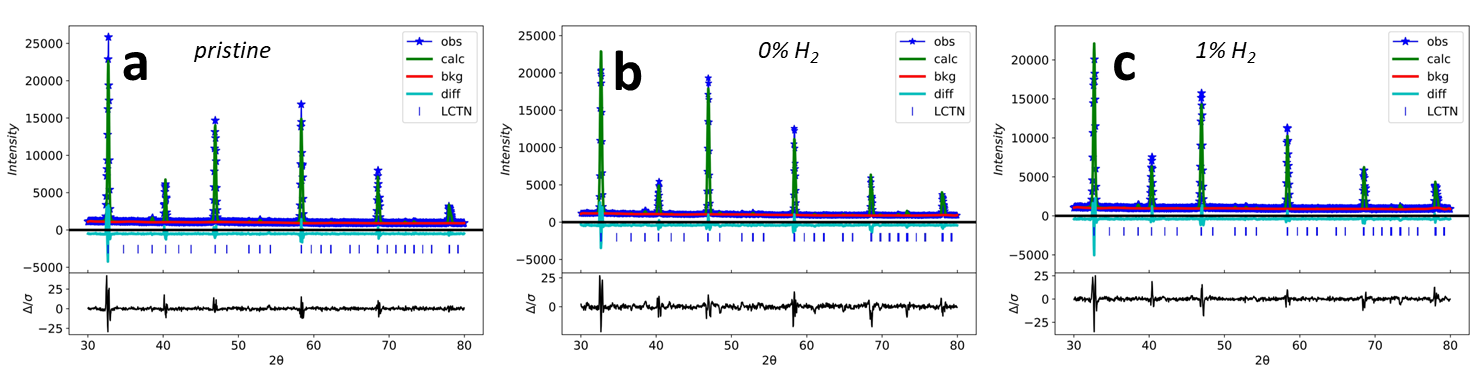


**Figure S28:** (a-c) Rietveld refinement of the XRD patterns of pristine, 0% H_2_ and 1% H_2_ LCTN samples respectively.

**Table S5:** Cell parameters obtained from the Rietveld refinement of LCTN samples.

**
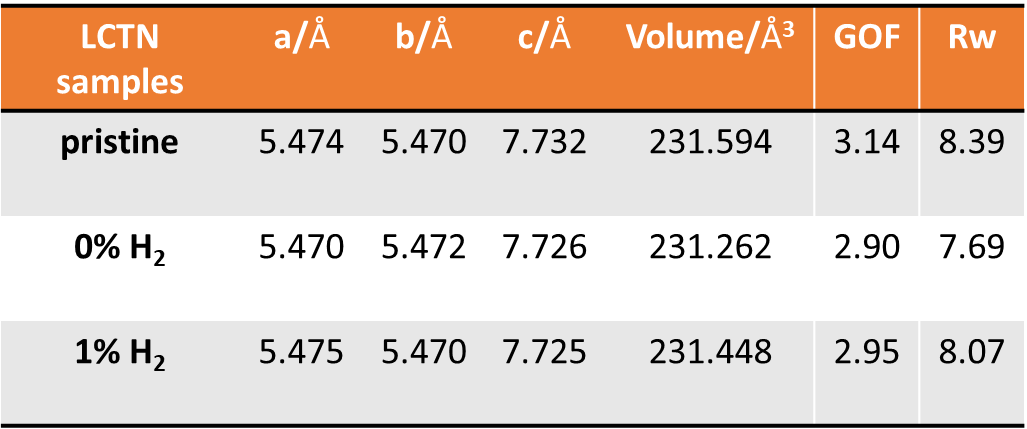
**

**References:**

(1) Schneider, C. A.; Rasband, W. S.; Eliceiri, K. W. NIH Image to ImageJ: 25 Years of Image Analysis. *Nat. Methods* **2012**, *9* (7), 671–675. https://doi.org/10.1038/nmeth.2089.

(2) Li, J. P. H.; Zhou, X.; Pang, Y.; Zhu, L.; Vovk, E. I.; Cong, L.; Van Bavel, A. P.; Li, S.; Yang, Y. Understanding of Binding Energy Calibration in XPS of Lanthanum Oxide by: In Situ Treatment. *Phys. Chem. Chem. Phys.* **2019**, *21* (40), 22351–22358. https://doi.org/10.1039/c9cp04187g.

(3) Toby, B. H.; Von Dreele, R. B. GSAS-II : The Genesis of a Modern Open-Source All Purpose Crystallography Software Package. *J. Appl. Crystallogr.* **2013**, *46* (2), 544–549. https://doi.org/10.1107/S0021889813003531.
